# Assessment of false discovery rate control in tandem mass spectrometry analysis using entrapment

**DOI:** 10.1101/2024.06.01.596967

**Published:** 2024-06-04

**Authors:** Bo Wen, Jack Freestone, Michael Riffle, Michael J. MacCoss, William S. Noble, Uri Keich

**Affiliations:** 1 Department of Genome Sciences, University of Washington; 2 School of Mathematics and Statistics, University of Sydney; 3 Paul G. Allen School of Computer Science and Engineering, University of Washington

## Abstract

A pressing statistical challenge in the field of mass spectrometry proteomics is how to assess whether a given software tool provides accurate error control. Each software tool for searching such data uses its own internally implemented methodology for reporting and controlling the error. Many of these software tools are closed source, with incompletely documented methodology, and the strategies for validating the error are inconsistent across tools. In this work, we identify three different methods for validating false discovery rate (FDR) control in use in the field, one of which is invalid, one of which can only provide a lower bound rather than an upper bound, and one of which is valid but under-powered. The result is that the field has a very poor understanding of how well we are doing with respect to FDR control, particularly for the analysis of data-independent acquisition (DIA) data. We therefore propose a new, more powerful method for evaluating FDR control in this setting, and we then employ that method, along with an existing lower bounding technique, to characterize a variety of popular search tools. We find that the search tools for analysis of data-dependent acquisition (DDA) data generally seem to control the FDR at the peptide level, whereas none of the DIA search tools consistently controls the FDR at the peptide level across all the datasets we investigated. Furthermore, this problem becomes much worse when the latter tools are evaluated at the protein level. These results may have significant implications for various downstream analyses, since proper FDR control has the potential to reduce noise in discovery lists and thereby boost statistical power.

## 1 Introduction

Controlling the false discovery rate (FDR) in mass spectrometry proteomics—that is, controlling the expected proportion of false positives among the set of reported proteins, peptides, or peptide-spectrum matches (PSMs)—is easy to get wrong. Most widely used FDR control procedures in proteomics involve target-decoy competition (TDC) where the observed spectra are searched against a bipartite database comprised of real (“target”) and shuffled or reversed (“decoy”) peptides [6]. Although this procedure can be rigorously proven to control the FDR in a spectrum-centric search, subject to several reasonable assumptions [11], in practice many analysis pipelines implement variants of the procedure that potentially fail to control the FDR. For example, PSM-level control using TDC is inherently problematic [11, 21]. Similarly, most pipelines involve training a semi-supervised classification algorithm, such as Percolator [12] or PeptideProphet [13], to re-rank peptide-spectrum matches (PSMs), which in practice can compromise FDR control [8].

Failure to correctly control the FDR can have serious negative implications. Most obviously, if a given analysis pipeline underestimates the empirical error rate in a particular experiment, which is known as the “false discovery proportion” (FDP)—i.e., if the pipeline claims that the FDR is controlled, say, at 1% but the actual average of the FDP is 5%—then the scientific conclusions drawn from those results may be invalid. Perhaps more insidiously, invalid FDR control can also impact our choice of analysis pipelines and make comparison of instrument platforms and proteomics workflows impossible. To see why this is the case, consider a hypothetical tool that consistently fails to control the FDR. In a benchmarking experiment, if we compare the number of proteins detected by a collection of analysis tools, all using a fixed FDR threshold, then the liberally biased tool will have a clear (and unfair) advantage.

To address this concern, therefore, it is important to have a rigorous procedure to evaluate the validity of the FDR estimates reported by a proteomics analysis pipeline. The standard way to carry out such an evaluation is via an “entrapment” procedure, in which the tool is provided with a combined target+entrapment database containing two types of sequences: the original standard set of target sequences that are derived from the proteome of the species that was used in the experiment, and a collection of “entrapment sequences” that do not appear among the original target sequences [10]. The underlying assumption of entrapment procedures is that any reported entrapment sequence is false and can therefore be used to estimate the proportion of false discoveries. Critically, the distinction between the two parts of the target+entrapment database is hidden from the tool itself, and is revealed only after the tool’s analysis is completed in order to evaluate the validity of the tool’s FDR control.

While conceptually simple, correctly carrying out an entrapment analysis can be tricky. Indeed, although the general idea of entrapment is widely used in the evaluation of tools for mass spectrometry proteomics analysis [2, 4, 18, 20, 22–24, 26–29, 31, 34, 35, 37, 39–41], we find that in practice many of these applications of entrapment procedures are invalid. Unfortunately, this means that, in some cases, a tool that appears to control the FDR may not do so when the correct entrapment procedure is employed.

In this work, we expose common errors in existing entrapment estimation approaches, as well as propose a novel estimation method that allows more accurate evaluation of FDR control for mass spectrometry analysis pipelines. We then apply several entrapment estimation methods to gauge the level of FDR control of four widely used tools for the analysis of data-dependent acquisition (DDA) data (MS-GF+ [15], Tide [5], Sage [19], MSFragger [17]), and three tools for data-independent acquisition (DIA) data (DIA-NN [4], Spectronaut, and EncyclopeDIA [33]). We find that the DDA search tools generally seem to control the FDR at the peptide level, whereas none of the DIA search tools consistently controls the FDR at the peptide level across all the datasets we investigated. Furthermore, this problem becomes much worse when DIA tools are evaluated at the protein level. These results suggest an opportunity for the field: insofar as existing methods yield results with unexpectedly high levels of noise, we anticipate that reducing this noise by accurately controlling the FDR has the potential to yield better statistical power in downstream analyses.

## 2 Results

### 2.1 Many published studies use entrapment incorrectly

**Figure 1:**
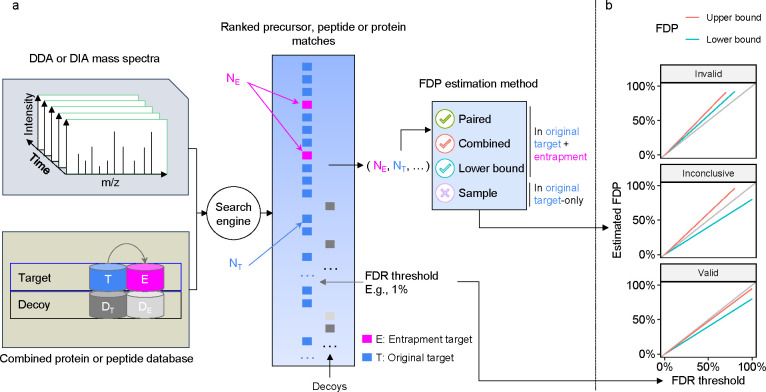
Entrapment strategy for FDR control evaluation. **(a)** Schematic of an entrapment method. The target database is augmented with entrapment sequences, and the augmented database is used by the search tool to produce a ranked list of peptides. In this example, we show the decoys that the tool is using to control the FDR, but the tool can use other methods to control the FDR. The target/entrapment labels are hidden from the search engine but are revealed to the entrapment method, allowing it to provide an estimated FDP. Note that some entrapment estimation methods require additional inputs besides the count of the number of original and entrapment targets. **(b)** Comparing the FDR reported or used by a given analysis tool (x-axis) to the estimated upper bound (blue) and lower bound (red) on the FDP produced by two different entrapment estimation methods allows us to conclude that the tool’s FDR estimates are valid (bottom) or invalid (top). If the bounds fall on either side of the line y=x (middle), then the analysis is inconclusive.

Before describing various methods for entrapment analyses, it is important to distinguish between methods that provide estimated upper bounds versus lower bounds of the FDP, and understand their limitations. The primary output of an entrapment procedure can be summarized by plotting the entrapment-estimated FDP as a function of the FDR cutoff used (or reported as a q-value) by the evaluated tool. If the entrapment procedure provides an estimated upper bound on the FDP, then the entrapment analysis suggests that the actual FDP falls below the observed line. Conversely, the entrapment procedure may provide a lower bound, indicating that the actual FDP falls above the line. Therefore, applying both an upper bounding and a lower bounding entrapment procedure to a given analysis tool can yield one of three outcomes (Figure 1): (1) if both procedures produce curves that fall below the line y=x, then we can take this as empirical evidence suggesting that the tool successfully controls the FDR; (2) conversely, if both curves fall above y=x, then we can use it as evidence suggesting that the tool fails to control the FDR; (3) if the estimated upper bound is above y=x and the lower bound is below y=x, then the experiment is inconclusive.

An important caveat to all of these analyses is that an entrapment procedure aims to gauge the FDP among the discoveries reported by the tool; however, the tool itself is typically designed to control the FDR, which is the expected value of the FDP. Hence, we should use reasonably large datasets in our entrapment analysis, where the FDP is typically close to the FDR (by the law of large numbers), or we should average the empirical FDP over multiple entrapment sets, which ameliorates the problem. Even in those cases, the random nature of the FDP implies that some deviations above y=x may be acceptable if they are small and rare.

With that caveat in mind, note that from a statistical perspective, the onus falls on the tool developer to establish that the method indeed controls the FDR, so “inconclusive” (scenario 3 above) is a tentative strike against the tool. Furthermore, FDR control should be universal; consequently, a valid FDR control procedure should achieve scenario (1) for any reasonably large dataset.

We next present the three main approaches in the literature to estimating the FDP. The first two methods aim to estimate the FDP in the combined, target+entrapment, list of original target and entrapment discoveries, whereas the third method aims to estimate the FDP in a stricter list, focusing only on the original target discoveries and excluding the entrapment discoveries. All three estimation approaches adjust their estimation based on a parameter r which measures the effective ratio of the entrapment to original target database size.

The first method, which we refer to as the “combined” method, estimates the FDP among the target+entrapment $\left(𝒯\;\cup \;{ℰ}_{𝒯}\right)$ discoveries as

(1) $${\hat{FDP}}_{𝒯\;\cup \;{ℰ}_{𝒯}}\;=\;\frac{N_{ℰ}(1\;+\;1/r)}{N_{𝒯}\;+\;N_{ℰ}},$$

where $N_{𝒯}$ and $N_{ℰ}$ denote the number of target and entrapment discoveries respectively. Equation (1) provides an estimated upper bound because this method tends to overestimate the true FDP (Supplementary Note S1). Thus, the combined method can be used to provide empirical evidence that a given tool successfully controls the FDR among its discoveries (Figure 1b). Notably, with r=1 Equation (1) reduces to Elias and Gygi’s original estimation of the FDR in the concatenated target-decoy database, which in our case is the target+entrapment database [6]. This combined method has been used in several previous studies (Table 1); for example, The *et al*. used it to evaluate the “picked protein” method for FDR control [37]. It has also been used to evaluate the DDA analysis tool Mistle [27], as well as for evaluation of the O-Pair method for gycloproteomics data [23].

Unfortunately, the combined estimation is often applied incorrectly to establish FDR control after removing the 1/r term:

(2) $${\hat{\underline{FDP}}}_{𝒯\;\cup \;{ℰ}_{𝒯}}\;=\;\frac{N_{ℰ}}{N_{𝒯}\;+\;N_{ℰ}}.$$

The problem is that without the 1/r term the estimate in Equation (2) becomes a lower bound on the FDP (Supplementary Note S1). As such, this method can only be used to show that a tool fails to control the FDR (Figure 1b), rather than as evidence of FDR control. In what follows we refer to Equation (2) as the “lower bound.” Table 1 shows that multiple studies incorrectly used the lower bound to validate FDR control, including a recent benchmarking study to evaluate several widely used DIA tools for proteomics and phosphoproteomics DIA data analysis [22]. In that review, the lower bound was used both correctly to point out questionable FDR control, as well as incorrectly as evidence of FDR control.

The third approach, which we refer to as the “sample” estimation method, estimates the FDP only among the original target (𝒯) discoveries as

(3) $${\hat{FDP}}_{𝒯}\;=\;\frac{N_{ℰ}\;\cdot \;1/r}{N_{𝒯}}.$$

This approach has been employed to evaluate DIA-NN [4], MSFragger-DIA [40], MaxDIA [34], and DIAmeter [24]. We argue that the sample entrapment method is inherently flawed because, while typically underestimating the FDP, it can also overestimate it in some unusual cases (Supplementary Note S2). Hence, the sample estimation method cannot be used to provide empirical evidence that a tool controls the FDR nor that the tool fails to control the FDR.

In addition to employing different methods to estimate the FDP, published studies also differ with respect to how the entrapment database is constructed. In the “shuffled entrapment” approach, the entrapment sequences are derived analogously to decoy sequences by shuffling the corresponding target sequences, whereas in the “foreign entrapment” approach they are taken from the proteome of some other species [10]. In this work, we primarily employ the shuffled entrapment approach, highlighting potential pitfalls of using foreign entrapment sequences in Supplementary Note S3 and Supplementary Figures S1–S4.

Our literature review, summarized in Table 1, indicates that many publications fail to use entrapment correctly. Overall, we identified only three studies that correctly use entrapment, and all three focused on DDA analysis [23, 27, 37]. A common mistake is to use the lower bound method, which cannot establish that a given method correctly controls the FDR [18, 22, 31, 39, 41], or to use the problematic sample entrapment method [4, 24, 34, 40]. Further discussion of some of the studies in Table 1 is provided in Supplementary Note S4.

### 2.2 The paired estimation method yields a tighter upper bound on the FDP

As noted above, the combined estimation method tends to overestimate the FDP and therefore provides an estimated upper bound. Indeed, we observed in practice that this method can often significantly overestimate the FDP, which motivated us to propose a complementary “paired estimation” approach that allows us to reduce this conservative bias by taking advantage of sample-entrapment pairing information. For this method to work, say, in peptide-level analysis, we require that each original target peptide be paired with a unique entrapment peptide (so in particular, r=1). In practice this means that the paired estimation method requires a shuffling or reversal to generate the entrapment peptides. Moreover, we assume that any target peptide that is not in the sample is equally likely to score higher than its paired entrapment peptide as the paired peptide is.

Given such paired entrapment peptides, and still considering peptide-level analysis, the paired method estimates the FDP in the list of target+entrapment discovered peptides by

(4) $${\hat{FDP}}_{𝒯\;\cup \;{ℰ}_{𝒯}}^{*}\;=\;\frac{N_{ℰ}\;+\;N_{ℰ\geq s>𝒯}\;+\;2\cdot N_{ℰ>𝒯\geq s}}{N_{𝒯}\;+\;N_{ℰ}},$$

where s is the discovery cutoff score; $N_{ℰ\geq s>𝒯}$ denotes the number of discovered entrapment peptides (scoring ≥s) for which their paired original target peptides scores < s; and $N_{ℰ>𝒯\geq s}$ is the number of discovered entrapment peptides for which the paired original target peptides scored lower but were still also discovered. In Supplementary Note S5 we explain the rationale behind this estimation method and show how it can be generalized to larger entrapment databases where each target peptide is uniquely associated with *k* entrapment ones (so r=k).

### 2.3 Comparing the estimation methods using data from a controlled experiment

We first demonstrate the qualitative differences among the above estimation methods—lower bound, sample, combined, and paired—using the ISB18 dataset, which consists of DDA data generated from a known mixture of 18 proteins [16]. We used the Tide search engine [5] to carry out FDR control at the peptide level (Section 4.1.2). Due to the relatively small size of the ISB18 dataset, we averaged each entrapment method’s estimated FDP over multiple applications, each with different randomly drawn decoy and entrapment databases. Accordingly, here we are comparing the empirical FDR rather than the FDP of the methods.

**Figure 2:**
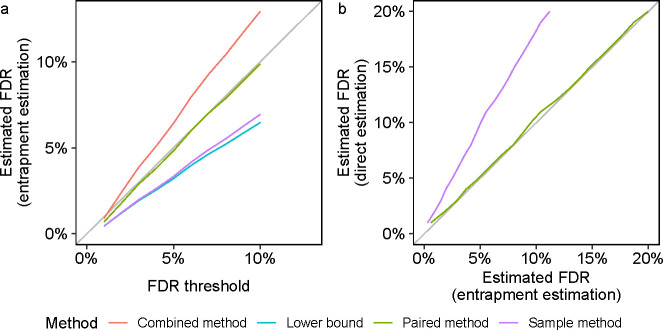
Entrapment analysis using the ISB18 data. The figure summarizes two different types of entrapment experiments, both using shuffled sequences with r=1. The lists of discoveries for both panels were generated by searching with Tide, followed by peptide-level FDR control. **(a)** The entrapment-estimated FDP in the reported list of target peptides (averaged over multiple decoys and entrapments) is plotted as a function of the given FDR threshold, for four different entrapment methods. The original target database consists only of the ISB18 peptides. **(b)** The FDP estimated by the paired and the sample entrapment estimation methods (averaged over multiple decoys and entrapments) is plotted against the corresponding castor-based estimates. The original target database consisted of the ISB18 and castor peptides at a ratio of 1:636.

In the first experiment, the original target database consists of the ISB18 peptides, and the entrapment part consists of shuffled sequences with r=1. We first focus on the paired and combined methods, both of which are estimating the FDP in the same list of target+entrapment discoveries at the given FDR threshold. Notably, the paired estimation method yields a curve that is below (or just at) the line y=x (Figure 2a). In contrast, the curve corresponding to the combined estimation method is distinctly above the diagonal, demonstrating the upper bound (conservative) nature of this method. In particular, in this example we can use the paired method to argue that the FDR seems to be controlled in this case (as we expect it to be), but we cannot make that argument using the combined estimate. As expected, the lower bound curve is below the diagonal but, as such, it is uninformative in this case. Finally, the fact that the sample method is also below the diagonal indicates that, like the lower bound, it is probably underestimating the true FDP here.

In the second experiment, we aimed to generate empirical evidence to support the theoretical arguments laid out in Supplementary Notes S2 and S5. In this experiment we took advantage of the controlled nature in which the ISB18 dataset was generated to conduct a double entrapment experiment, which is designed to gauge how accurately the sample and paired entrapment methods estimate the FDP. Specifically, we constructed an extended “original target” database that consisted of the ISB18 peptides augmented by a much larger set of peptides from a foreign species (the castor bean). The ratio of ISB18 to castor peptides in this new original target database is 1:636. We then applied the paired and sample entrapment methods using shuffled sequences (r=1) to estimate the FDP among the reported peptides. The controlled nature of the ISB18 dataset implies that any reported castor peptide is a false discovery. At the same time, with a ratio of 1:636 ISB18-to-castor peptides it is reasonable to assume that any ISB18 reported peptide is a true discovery. This setup allows us to directly estimate the FDP in each discovery list, which we can then compare to the estimate produced by the entrapment procedures.

The results of this experiment (Figure 2b) provide further evidence supporting the validity of the paired method: the FDR in the target+entrapment set of discoveries estimated by the paired method essentially agrees with the “direct” castor-based estimate, where the latter counts every entrapment or castor peptide as a false discovery. In contrast, the sample entrapment seems to substantially underestimate the castor-based estimate because it is trying to estimate the FDP in the wrong list of discoveries — the ISB18+castor ones — ignoring the fact that the tool was instructed to control the FDR in the larger set of ISB18+castor+shuffled entrapments.

### 2.4 DDA search tools largely appear to control the FDR at the peptide level

Next we set out to use these entrapments methods to compare the FDR control provided by four different DDA search engines—Tide, Sage, MS-GF+ and MSFragger—using data generated from a complex sample, rather than from a controlled mixture. For MS-GF+ we carry out peptide-level FDR control using the primary search engine scores, whereas for the other three search engines we use a machine learning post-processor: Percolator-RESET for Tide, Sage’s built-in linear discriminant analysis, MSBooster [38] for MSFragger. For this analysis, we use data from 24 MS/MS analyses of the human cell line HEK293, searched against target+entrapment databases for which the human reference proteome was taken as the original target sequences, and paired shuffled entrapment sequences (so r=1). Thus, in addition to being derived from a more complex sample, this dataset is substantially larger than the ISB18 dataset, as is the original target database. Having established that the sample estimation method is inherently problematic, from here onward we consider only the lower bound, combined, and paired estimation procedures.

**Figure 3:**
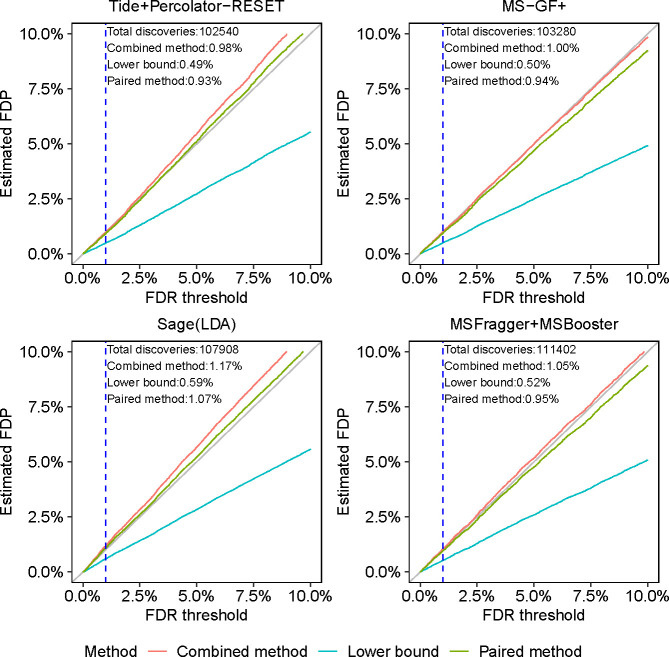
Comparing entrapment procedures using HEK293 DDA data. Each figure shows, for a given search procedure, the (shuffled with r=1) entrapment-estimated FDP in the list of target peptides that was reported at the given FDR threshold. The dashed vertical line is at the 1% FDR threshold, as are the numbers reported in text in the figure.

*A priori*, given the established nature of DDA FDR analysis, we expect all of these tools to produce valid FDR estimates. Accordingly, the conclusions we draw from the analysis of the HEK293 data (Figure 3) largely mirror those we drew from the Figure 2a. Specifically, for all peptide-level analysis tools the paired method yields estimated FDPs that are quite close to the diagonal. The more conservative nature of the combined method is on display again: it is always above the paired estimation curve. More specifically, relying on the combined method we cannot claim we have evidence that Tide+Pecolator-RESET and Sage control the FDR in this setup, but we can argue for such apparent evidence if we use the paired method. As expected, the lower bound seems to consistently significantly underestimate the FDP.

### 2.5 DIA search tools fail to consistently control the FDR at the peptide level, and the problem is much worse at the protein level

Turning to tools for analysis of DIA data, we performed a more extensive evaluation. Specifically, we applied three different search engines—DIA-NN, Spectronaut and EncyclopeDIA—to the ten datasets listed in Table 3. In the case of EncyclopeDIA, we only analyzed the four datasets for which we have gas phase fractionation runs. In each case, we applied all three entrapment estimation methods separately at the precursor (for DIA-NN and Spectronaut) or peptide (for EncyclopeDIA) level and at the protein level for each search engine. Note that, because Spectronaut only estimates precursor-level FDR for each run, rather than for a full experiment, one MS run from each DIA dataset was used and we only report precursor-level results for a selected single MS run for each dataset.

**Figure 4:**
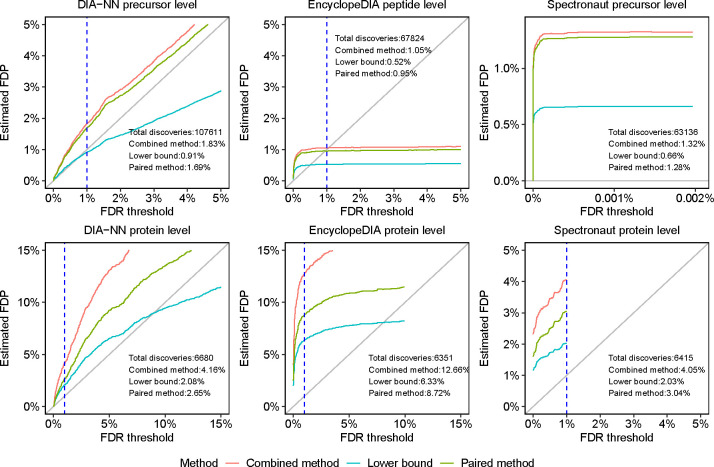
Entrapment evaluation of the FDR control of DIA analysis tools. DIA-NN, EncyclopeDIA and Spectronaut were applied to the human-lumos DIA dataset using shuffled entrapment with r=1. The top row shows the precursor or peptide-level estimated FDPs, and the bottom row shows the corresponding protein-level analysis. In the Spectronaut precursor-level plot, the x axis was set to show the maximum FDR threshold reported by the tool, which was less than the 1% threshold set in the analysis. The dashed vertical lines are at the 1% FDR threshold, as are the numbers reported in text in the panels.

The results of this experiment suggest that the precursor or peptide-level FDR control is frequently questionable, whereas protein-level FDR control is frequently invalid (Table 2). For example, for the human-lumos dataset (Figure 4) we observe that the precursor or peptide-level FDR control appears to be inconclusive, whereas the protein-level FDR control is apparently invalid: e.g., the lower bound on EncylopeDIA’s FDP is above 6%. Indeed, keeping in mind that the true FDP is likely to be between the lower bound and the paired-estimated upper bound, we see that the protein-level 1% FDR control appears to be consistently invalid for EncyclopeDIA and Spectronaut and mostly invalid for DIA-NN. Although the peptide-level analysis indicate substantially better control of the FDR, there are still some datasets for which the results are inconclusive or worse. Notably, this is particularly the case for the single cell (1cell-eclipse) data, where the lower bound on DIA-NN’s precursor-level FDP data is above 2.3% and Spectronaut’s FDP is above 3.8%. It is worth noting that DIA-NN’s and Spectronaut’s protein-level estimated FDPs are also highest on that particular data set. Supplementary Figures S5–S10 complement these observations by providing for all datasets and DIA-tools the lower bound, as well as the combined and paired estimated FDPs for a wide range of FDR thresholds. In particular, the figures consistently demonstrate how the entrapment estimation methods compare with one another, with the combined method reporting the largest estimated FDP, the lower bound the smallest, and the paired method in between the other two.

We also performed complementary experiments in which we varied the entrapement-to-original-target ratio r. Consistent with Lou *et al.* [22], we find that the estimated FDP among DIA-NN’s reported proteins generally increases with r (Supplementary Figure S11–S12). On the other hand, Lou *et al.* only increased r up to r≈1 and concluded that DIA-NN controls the FDR (because they were incorrectly using the lower bound, Section S4). In contrast, we explored higher values of r and found that even the lower bound is consistently substantially higher than the corresponding FDR thresholds, indicating quite clearly that DIANN apparently fails to control the FDR in those scenarios. For example, for r=8 we see a lower bound of almost 7% (Supplementary Figure S11). The same phenomenon was observed in the case of Spectronaut as well: for example, the lower bound is above 4% for r=6 (Supplementary Figure S13). While these observations do not imply that the FDP is as high when searching only the original target database, they do indicate that the tool struggles to control the FDR in some setups. In general, proper FDR control should be applicable to any realistic scenario.

## 3 Discussion

Overall, our analysis seems to suggest that the application of entrapment methods for evaluating FDR control in proteomics analysis is quite challenging. Indeed, our literature survey suggests that, in practice, a variety of estimation methods have been used to evaluate both DDA and DIA search tools, some of which are invalid as either a lower bound or as an upper bound estimate, while others are often incorrectly used. Thus, our goal here is to clarify the apparent confusion about entrapment estimation methods while offering a new and improved estimation method. To facilitate future entrapment analyses, we have produced an open source software tool, FDRBench, that provides two main functions: (1) build entrapment databases using randomly shuffled target sequences or using sequences from foreign species, and (2) estimate the FDP using the lower bound, combined, and paired methods.

Our discussion of the validity of the entrapment estimation methods implicitly relies upon the same assumptions that guarantee the applicability of TDC. In particular, the discussion cannot overcome unrealistic or poorly constructed entrapment sequences, which brings us to the question of the entrapment construction. In this work, we chose to focus on shuffled entrapments because, as we argue in detail in Supplementary Note S3 and Supplementary Figures S1–S4, using the alternative of foreign species raises complex questions associated with the choice of those species; for example, what is the “right” evolutionary distance for the entrapment species, and whether the entrapment species coincides with a potential source of contamination, as we found in our analysis of the commonly used HEK293 dataset. Such questions will need to be addressed before we can objectively agree on a way to use such foreign entrapment sequences.

Majed and Lam recently argued that using randomly shuffled entrapment sequences when the decoys are generated through random shuffling amounts to circular reasoning [25]. We only partially agree with this statement. Specifically, we contend that this reservation is valid only if the analysis tool’s use of the decoy sequences is restricted to estimating the FDR using the same estimation method that the entrapment relies on. However, both the lower bound and our new paired estimation method do not use the entrapments in the same way that TDC uses its decoys. Moreover, the analysis tool often relies on the decoys in a more sophisticated manner that might be problematic. For example, Percolator’s cross-validation scheme to improve the ranking of the PSMs can inadvertently misuse the target/decoy label when multiple spectra are generated from the same peptide species [8]. Because in this case the compromised FDR control stems from indirectly peeking at the target/decoy label, the problem can be identified even when both the entrapment sequences and the decoys are shuffled.

As mentioned, in general an upper bound estimate like the combined estimation can only be used to show valid FDR control, and the lower bound to highlight invalid FDR control. However, if r is large then the difference between Equations (1) and (2) becomes negligible, so each of the methods can be reasonably used for making both arguments. That said, keep in mind that using r≫1 creates creates a much larger combined target database, most of which is made of entrapment sequences. Thus, establishing FDR control in this somewhat atypical setup is not as convincing as establishing it for smaller values of r (e.g., r=1,2). This is where the paired entrapment estimation method, Equation (4), and its k-matched generalization, Equation (S1), which provide tighter upper bounds, can become particularly useful.

Our entrapment experiments using DIA analysis tools show that their FDR control at the protein level is often questionable. Given the importance of protein-level analysis to mass spectrometry experiments, we believe that this result should serve as a call to further research into this problem. Keep in mind that this is not just a matter of rigorous statistics: often the detected proteins are then further used to identify differentially expressed proteins, so incorrectly calling the detected proteins can adversely affect our ability to successfully accomplish our ultimate goal.

## 4 Methods

### 4.1 TDC protocols for controlling the FDR

#### 4.1.1 TDC

Initially introduced by Elias and Gygi [6], the following variation of the original TDC procedure was subsequently proved to control the FDR [11]: the input is a list of pairs $\left(W_{i},\;L_{i}\right)$, where $L_{i}\;=\;+1$ for a target PSM, peptide, or protein, $L_{i}\;=\;-1$ for a decoy, and $W_{i}$ is its corresponding score.

The pairs are ordered in decreasing order of their score $W_{i}$, and for each k we find $D_{k}$, the number of decoys in the top k pairs, and $T_{k}\;=\;k\;-\;D_{k}$ the number of targets. Given the desired FDR level α, TDC sets the rejection/discovery index at

(5) $$K\;=\;K\left(\alpha \right)\;≔\;max\left\{k\;:\frac{D_{k}\;+\;1}{max\left\{T_{k},\;1\right\}}\;\leq \;\alpha \right\}.$$

Finally, TDC reports all targets among the top K pairs.

Assuming that a pair corresponding to an incorrect discovery is at least as likely to be a decoy as it is to be a target, i.e., that $P\left(L_{i}\;=\;-1\right)\geq 1/2$ independently of everything else (including of the score $W_{i}$), TDC rigorously controls the FDR [1, 11] among its reported discoveries.

#### 4.1.2 Peptide-level TDC with PSM-and-peptide

PSM-and-peptide is a TDC-based procedure for peptide-level analysis introduced in [21]. This method involves a double competition: the first is a PSM-level competition where each spectrum is searched against the concatenated target-decoy database and the best matching peptide is assigned to it (where ties are broken randomly). This defines the (optimal) PSM associated with each spectrum, and then each target or decoy peptide is assigned a score, which is the maximum of the scores of all PSMs which this peptide is part of. PSM-and-peptide introduces a second level of competition to define the scores and labels by utilizing the pairing between each target and its shuffled decoy. Specifically, it keeps only the higher scoring peptide from each target-decoy pair. Any peptide with no matching PSM is assigned the lowest possible score, and all ties are randomly broken. This procedure defines the pair’s winning score $W_{i}$ and its label $L_{i}\;=\;\pm 1$ indicating whether the higher scoring peptide was the target or the decoy. TDC is then applied as above to this list of scores and labels (W,L).

### 4.2 Datasets

The MS/MS data used in this study include a wide range of datasets from different vendors, different MS instruments, data acquisition strategies, and species. As shown in Table 3, a total of 12 datasets were used in the study, including two DDA datasets and 10 DIA datasets. The raw data were downloaded from PRIDE, MassIVE and jPOST. The raw data were converted to mgf or mzML format files using MSConvert in ProteoWizard (version 3.0.24031) [3]. This excludes the ISB18 data, where ms2 files were obtained from [21]. Among the 10 DIA datasets, four include gas phase fractionation DIA runs, which were used to build chromatogram libraries for EncyclopeDIA analysis. Two of the datasets were from a previous single-cell proteomics study (Dataset ID: PXD023325).

### 4.3 Entrapment database generation using random sequences

The shuffled entrapment databases were generated differently for precursor-level and peptide-level FDR control evaluation than they were for protein-level analysis. In both cases, the original target protein sequences for human (UP000005640, 20597 proteins), yeast (UP000002311, 6060 proteins) and mouse (UP000000589, 21701 proteins) were downloaded from UniProt (02/2024).

For precursor/peptide-level analysis, the original target proteins were first *in silico* digested into peptides using trypsin (without proline suppression) with one missed cleavage allowed. The original target peptides database consisted of those with lengths between 7 and 35 amino acids. Then for each original target peptide, we attempted to generate a paired random entrapment peptide as follows. Specifically, the original peptide was shuffled while keeping the C-terminal amino acid fixed and then searched against all original target peptides as well as the previously generated random peptides to ensure it is distinct from all of those. If it was not, we retried to generate such a distinct shuffled peptide up to an additional 20 times. If all those attempts failed we removed the corresponding peptide from the original target database. To generate r matching random entrapment peptides we repeated this shuffling process up to 20+r times to try and obtain r distinct entrapment peptides for each original target peptide. A peptide for which we failed to generate r distinct shuffles after 20+r attempts was removed from the original target database.

In the protein-level FDR evaluation, for each original target protein we generated a paired random entrapment protein as follows. We first *in silico* digested the original protein into peptides using trypsin (without proline suppression). In this step, all the peptides, irrespective of length and mass constraints, were retained and no missed cleavages were considered. Then, for each of these peptides we tried to generate a distinct randomly shuffled entrapment peptide (again, while fixing the C-terminal) as above. This was tried up to 20 additional times for each original peptide and if all failed to generate a distinct peptide then (in contrast to the peptide-level analysis) the entrapment peptide associated with the target peptide was identical to the target. Finally, each original peptide in the considered protein was swapped with its randomly generated one. Note that a peptide that appears in multiple proteins or multiple times within a protein was consistently swapped with its uniquely associated paired entrapment peptide in all the proteins it appears. To create an r-fold entrapment protein database, we attempted to associate with each digested original target peptide r distinct randomly shuffled entrapment peptides as above. If we failed to do so in 20+r attempted shuffles then we randomly sampled with replacement r−n additional entrapment peptides from the n>0 distinct shuffles that we managed to generate. If there were no distinct shuffles at all (n=0) then the selected r entrapment peptides were all identical to the original digested peptides. We then used these r entrapment peptides to define r associated entrapment proteins as described above for r=1.

### 4.4 Entrapment database generation using sequences from foreign species

To generate protein-level entrapment databases using proteins from foreign species, a set of proteins from the selected foreign species were randomly selected as entrapment proteins to achieve the desired ratio of r entrapment-to-original target proteins (we used r≥1).

To generate peptide-level entrapment databases, both original target proteins and the proteins from the foreign species were *in silico* digested into peptides using trypsin (without proline suppression) with one missed cleavage allowed. Again, we only considered digested target and entrapment peptides with length between 7 and 35 amino acids. Any foreign species peptides that matched an original target peptide were removed. Finally, we randomly selected as many of the remaining foreign peptides as needed to achieve the desired ratio of r entrapment-to-(remaining)-original target peptides (we used r≥1).

Two sets of foreign species were used in this study. The first set of foreign species consisted of *Arabidopsis thaliana* and *Saccharomyces cerevisiae*. The protein sequences from these two species were downloaded from UniProt (02/2024). Specifically, 27448 proteins from *Arabidopsis thaliana* (UP000006548) and 6060 proteins from *Saccharomyces cerevisiae* (UP000002311) were used. The second set of foreign species consisted of *Macaca mulatta*, *Callithrix jacchus*, *Papio anubis* and *Mus musculus*. The protein sequences from these species were downloaded from UniProt (primates: 04/2024, mouse: 02/2024). Specifically, 21590 proteins from *Papio anubis* (UP000028761), 21893 proteins from *Macaca mulatta* (UP000006718), 22027 proteins from *Callithrix jacchus* (UP000008225) and 21701 proteins from *Mus musculus* (UP000000589) were used.

### 4.5 FDR control evaluation procedures

#### 4.5.1 Using the ISB18 controlled experiment data (with Tide)

We used DDA spectra from an 18-protein mixture, called the ISB18, acquired from a controlled experiment [16]. We implemented our entrapment methods using two different setups: (a) randomly shuffled entrapment sequences and (b) randomly shuffled entrapment sequences in the presence of foreign target sequences from the castor proteome. We used the 9 ms2 spectrum files and the castor proteome from [21] and directly downloaded the in-sample protein database 20130710-ISB18-extended.fasta from https://regis-web.systemsbiology.net/PublicDatasets/database.

Here we used the following variant of the shuffling entrapment protocol described in Section 4.3. For approach (a) we created 100 randomly shuffled databases by digesting the 18-protein database using the tideindex command (default settings) from a recent version of Crux (v4.1.6809338) [14] and randomly shuffling the resulting original target peptides 𝒯 while fixing both the C-terminal and the N-terminal amino acids in place. We implemented a narrow search of the combined spectrum files against each of the 100 combined target+shuffled entrapment databases using tide-search (using the automatic fragment and precursor tolerance selection). Next, each entrapment method was used by considering 3 of the narrow search files, designating 1 of the randomly shuffled databases as ${ℰ}_{𝒯}$ and the remaining 2 randomly shuffled databases as the decoys $𝒯\;\cup \;{ℰ}_{𝒯}$. A small number of randomly shuffled peptides were problematic because they appeared in two different set of $𝒯,\;{ℰ}_{𝒯}$, or the decoy set of ${ℰ}_{𝒯}$, and some low complex target peptides were unable to produce 3 distinct random shuffles. Hence, any target peptide and their corresponding shuffled peptides that contained such a problematic peptide were removed from the search files. Next, we joined the 3 search files and implemented peptide-level analysis using the PSM-and-peptide protocol with XCorr scores. Finally, we estimated the FDP using each of the entrapment methods. Because we have 100 randomly shuffled databases, we repeated the above analysis 100 times using a different choice of the 3 narrow search files, ensuring that each narrow search file is considered exactly 3 times in total. We then estimated the FDR by taking the average of our 100 FDP estimates.

For approach (b) we prepared a new “original target” peptide database, , by combining the target sequences digested from the ISB18 protein mixture and the foreign sequences digested from the castor proteome. We then followed the same steps in (a) to obtain 100 FDP estimations that were averaged to obtain an FDR estimate using the paired and sample entrapment methods. We also obtained 100 “direct FDP” estimates, which rely on the number of discovered shuffled entrapment sequences and castor peptides to estimate the number of false discoveries. Specifically, the direct estimate is the ratio of the number of castor and shuffled entrapment discoveries over the total number of (ISB18+castor+shuffled entrapment) discoveries. These FDP estimates were also averaged to obtain a “direct FDR” estimate.

#### 4.5.2 Tide

To evaluate the FDR control in Tide (within Crux v4.1.6809338) [5] in a more typical setting, the HEK293 DDA MS/MS data was analyzed using Tide. Specifically, a peptide-level paired entrapment database was first generated using the method described in Section 4.3. Tide-index from the Crux toolkit (https://crux.ms/) was then used to randomly shuffle both the original target peptides and entrapment peptides in the peptidelevel entrapment database to obtain decoys (while keeping the C-terminal amino acid fixed) and build an index later used for tide-search. Carbamidomethylation of cysteine was set as a fixed modification and no variable modification was used in this step. Next, the HEK293 MS/MS data was searched against the combined database generated in the previous step using tide-search from the Crux toolkit with the following parameters: tailor-calibration, enabled; Sp scoring, enabled; precursor ion mass tolerance, 20 ppm; fragment tolerance, 0.02 m/z; fragment offset, 0. All other Tide parameters were set as default. FDR control of the Tide search result at the peptide level was performed using the single-decoy Percolator-RESET (version 0.0.6) [7] with specifying the Tailor score [36] as the primary score (all other parameters were set as default).

#### 4.5.3 Sage

To evaluate the peptide-level FDR control in Sage (version 0.14.6) [19], we generated the same target+shuffled entrapment database as described above for Tide (Section 4.5.2). In addition, to demonstrate the evolutionary distance problem with foreign entrapment, we also used Sage with foreign peptide entrapments as described in Section 4.4. In both cases the HEK293 dataset was searched against the target+entrapment database using Sage with the following parameters: fixed modification, carbamidomethyl (C); no variable modifications; precursor ion mass tolerance, 20 ppm; fragment ion tolerance, 20 ppm; enzyme digestion was disabled; peptide length range, 7–35; isotope error range was set to “[0,0]”. All other parameters were set as default. Sage’s built-in FDR control procedure was used. The “peptide q” from Sage’s output was used as the peptide q-value for downstream analysis.

#### 4.5.4 MS-GF+

To evaluate the FDR control in MS-GF+ (version 2023.01.12) [15], a peptide-level entrapment database was used that contained sample peptides, paired entrapment peptides and their paired decoy peptides. The peptide-level entrapment database was generated using the method described in Section 4.3. Specifically, in generating the peptide database, three different random peptides were generated for each target peptide, one decoy peptide was taken as paired entrapment peptide for the target while the other two decoy peptides were taken as decoy peptides. The HEK293 dataset was searched against the entrapment database using the following parameters: fixed modification, Carbamidomethyl (C); no variable modifications; precursor ion mass tolerance, 20 ppm; range of allowed isotope peak errors, “0,0”; peptide length range, 7 – 35; instrument ID, 3 (Q-Exactive); fragmentation method, 3 (HCD); protocol ID, 5 (Standard); n-terminal methionine cleavage was disabled. No enzyme digestion was applied. The parameter “-tda” was set to 0 to allow using the decoy peptides contained in the peptide database. All other parameters were set as default. The “PepQValue” from MS-GF+’s output was used as the peptide q-value for downstream analysis.

#### 4.5.5 FragPipe

To evaluate peptide-level FDR control of the FragPipe pipeline (version 21.1) [17, 38, 40] on DDA data, the HEK293 dataset was searched against the same peptide entrapment database used in the MS-GF+ peptidelevel FDR control analysis. The “Default” workflow setting in FragPipe was used with several parameters changed as follows. The enzyme digestion was configured to disable *in-silico* digestion. The setting of “Clip N-term M” was disabled. No variable modification was used. The isotope error was set to zero. The calibration and optimization setting was disabled. The “pin” files generated by MSBooster [38] were combined and used for Percolator (version 3.6.4) [12] analysis. The peptide-level result from Percolator was then used for downstream analysis.

For protein-level FDR control analysis on DDA data, the “Default” workflow setting in FragPipe. The setting of “Clip N-term M” was disabled.

#### 4.5.6 DIA-NN

For precursor-level FDR control evaluation of DIA-NN (version 1.8.1) [4], a peptide-level entrapment database was used that contained sample peptides and paired entrapment peptides for each DIA dataset. Each peptidelevel entrapment database was generated using the method described in Section 4.3. DIA-NN analysis was performed using the following parameters: fixed modification, carbamidomethyl (C); no variable modifications; enzyme digestion was disabled; peptide length range, 7–35; precursor charge range, 2–4. The setting of “N-term M excision” was disabled. The precursor FDR was set to 10%. All other parameters were set to their default values. For single run DIA data, the “Q.Value” from the main report was used as precursor q-value for downstream analysis. For datasets with multiple runs, the “Lib.Q.Value” from the main report was used as precursor q-value for downstream analysis.

For protein-level FDR control evaluation, we ran DIA-NN in its library-free mode using an entrapment database as described in Section 4.3. The enzyme and peptide length settings were the same as peptidelevel entrapment database generation in the precursor-level FDR control evaluation. The precursor FDR threshold was set to 1%. All other parameters were set as the same with the precursor-level analysis. For single run DIA data, the “PG.Q.Value” from the main report was used as protein q-value for downstream analysis. For datasets with multiple runs, the “Lib.PG.Q.Value” from the main report was used as protein q-value for downstream analysis.

#### 4.5.7 EncyclopeDIA

We evaluated both the peptide-level and the protein-level FDR control of the gas phase fractionated (GPF) chromatogram library analysis workflow with EncyclopeDIA (version 2.12.30) [32, 33].

We first used Oktoberfest (version 0.6.2) with Prosit models (fragment ion intensity prediction model: Prosit 2020 intensity HCD, retention time model: Prosit 2019 irt) [9, 30] to generate two *in silico* spectral libraries for each DIA dataset that were later used for EncyclopeDIA analysis. In the spectral library generation step, carbamidomethyl of cysteine was considered as a fixed modification, and no variable modifications were considered. Precursor charges 2 to 4 were considered. The normalized collision energy (NCE) parameter was set to 27. The first spectral library was used for peptide-level FDR control analysis in which the input to Oktoberfest for library generation was a peptide database in csv format containing the target peptides and their paired entrapment peptides as described in Section 4.3. The second spectral library was used for protein-level FDR control analysis in which the input to Oktoberfest for library generation was a protein database containing the target proteins and their paired entrapment proteins as described in Section 4.3. For the second library generation for each DIA dataset, trypsin (without proline suppression) with one missed cleavage allowed was used and only peptides with lengths between 7 and 35 amino acids were considered in Oktoberfest.

Next, for each FDR control evaluation analysis (peptide level or protein level), we generated a new spectral library by searching a set of GPF library DIA runs against the corresponding *in silico* spectral library using EncyclopeDIA.

Finally, we searched quant DIA runs against the GPF-derived spectral library in each FDR control evaluation analysis. In the analysis, the V2 scoring of EncyclopeDIA was enabled except for the TripleTOF 5600 dataset (PXD028735, human-tripletof). For protein-level FDR evaluation, the protein FDR threshold in the quant DIA analysis was set to 10% and the peptide FDR threshold was set to 1%. For peptide-level FDR evaluation, the protein FDR threshold in the quant DIA analysis was set to 1% and the peptide FDR threshold was set to 10%. The EncyclopeDIA analysis was run through the nf-skyline-dia-ms workflow (https://nf-skyline-dia-ms.readthedocs.io).

#### 4.5.8 Spectronaut

We evaluated both the precursor-level and the protein-level FDR control of the library-free analysis workflow (directDIA) in Spectronaut (version 18.7.240325.55695).

For evaluating the protein-level FDR control we constructed a protein database containing the original target proteins and their paired entrapment proteins as described in Section 4.3. We used Spectronaut with the following settings: enzyme specificity, trypsin (without proline suppression); maximum missed cleavages, 1; peptide length range, 7 – 35; toggle N-terminal M, disabled. All other parameters were set as default. We used the “PG.Qvalue” from Spectronaut output as the protein q-value for downstream analysis.

For evaluating the precursor-level FDR control we constructed a peptide database containing the target peptides and their paired entrapment peptides as described in Section 4.3. Because Spectronaut only estimates precursor-level FDR for each run, this analysis was done using only one MS run from each DIA dataset. No variable modification was set and no enzyme digestion was applied. The peptide length range was set from 7 to 35; toggle N-terminal M was disabled. In addition, the precursor PEP cutoff, protein q-value cutoff (experiment and run), protein PEP cutoff were set to 0.99. All other Spectronaut parameters were set as default. We used the “EG.Qvalue” from Spectronaut output was used as the precursor q-value for downstream analysis.

### 4.6 Code availability

We implemented the entrapment database generation methods as well as the different FDP estimation methods in a Java tool called FDRBench. The source code is available with an Apache license at https://github.com/Noble-Lab/FDRBench.

## Supplementary Material

Supplement 1

## Figures and Tables

**Table 1:** Summary of previous entrapment analyses.

Citation	Tools analyzed	DIA	DDA	Entrapment method	Entrapment composition	Valid?

Peckner 2018 [28]	Specter	✓		Other	foreign	
Demichev 2020 [4]	DIA-NN	✓		Sample	foreign	
Lu 2020 [23]	O-Pair		✓	Combined*	foreign	✓
Sinitcyn 2021 [34]	MaxDIA	✓		Sample	foreign	
Lu 2021 [24]	DIAmeter	✓		Sample	shuffled	
The 2022 [37]	PPGF		✓	Combined*	shuffled	✓
Lee 2022 [20]	cTDS		✓	Sample	foreign	
Na 2022 [26]	Deephos		✓	Sample	foreign	
Ye 2023 [39]	DIA-NN, Spectronaut	✓		Lower bound	shuffled	
Lancaster 2023 [18]	Spectronaut	✓		Lower bound	foreign	
Scott 2023 [31]	GPS	✓		Lower bound	foreign	
Nowatzky 2023 [27]	Mistle		✓	Combined	foreign	✓
Yu 2023 [40]	MSFragger-DIA, DIA-NN	✓		Sample	foreign	
Penny 2023 [29]	Spectronaut	✓		Sample	foreign	
Zhang 2023 [41]	Mzion		✓	Lower bound	foreign	
Lou 2023 [22]	Benchmarking	✓		Lower bound	foreign	
Strauss 2024 [35]	AlphaPept		✓	Other	foreign	
Bubis 2024 [2]	Spectronaut	✓		Sample	shuffled	

“PPGF” is the picked protein group FDR method. The final column indicates whether the entrapment method is deemed invalid to demonstrate FDR control.

* Lu et al. 2020 and The *et al.* 2022 employed large entrapment databases for the combined method.

**Table 2:** Entrapment analysis of FDR control of three DIA analysis tools.

Dataset	DIA-NN		EncyclopeDIA		Spectronaut	
	Precursor	Protein	Peptide	Protein	Precursor	Protein

human-astral	0.7–1.3 (?)	1.5–2.1 (X)			0.8–1.5 (?)	1.6–2.3 (X)
human-qe	0.7–1.3 (?)	1.5–2.2 (x)	0.7–1.3 (?)	4.3–6.4 (X)	0.7–1.4 (?)	1.3–1.9 (X)
human-tripletof	0.6–1.1 (?)	1.0–1.5 (X)	0.8–1.5 (?)	2.5–3.7 (X)	0.7–1.4 (?)	1.5–2.8 (X)
yeast-lumos	0.6–1.0 (✓)	1.2–1.4 (X)	0.4–0.7 (✓)	3.5–4.1 (X)	0.8–1.5 (?)	1.3–1.6 (X)
mouse-qe	0.7–1.3 (?)	0.8–1.1 (?)			0.7–1.4 (?)	1.3–1.9 (X)
human-timstof2	0.6–1.1 (?)	0.8–1.2 (?)			0.7–1.5 (?)	1.4–2.1 (X)
human-timstof1	0.7–1.2 (?)	0.8–1.2 (?)			0.7–1.3 (?)	1.1–1.7 (X)
100cell-eclipse	0.9–1.6 (?)	1.3–1.8 (X)			0.9–1.7 (?)	1.8–2.9 (X)
1cell-eclipse	2.3–4.7 (X)	2.0–3.5 (X)			3.8–7.6 (X)	3.0–5.6 (x)
human-lumos	0.9–1.7 (?)	2.1–2.6 (X)	0.5–1.0 (✓)	6.3–8.7 (X)	0.7–1.3 (?)	2.0–3.0 (X)

Each entry in the table lists the lower bound and paired-estimated upper bound on the empirical FDP among the target+entrapment discoveries reported by the DIA search engine at an FDR threshold of 1%. The entrapment procedures used shuffled entrapment sequences with *r* = 1 and were applied at both the peptide or precursor level and the protein-level. Each entry is followed by an indicator for whether the FDR control for this tool on this dataset is deemed valid (✓), invalid (X), or inconclusive (?). EncyclopeDIA results are provided only for the four datasets with gas phase fractionation data available. Note that the evaluation of each method is based on the full results presented in Figure 4 and Supplementary Figures S5–S10.

**Table 3:** Summary of datasets used in the study.

Dataset ID	Dataset name	MS instrument	MS runs	Species	Data type

ISB18	ISB18	LTQ Orbitrap	9	various	DDA
PXD001468	HEK293	Q-Exactive	24	human	DDA
PXD042704	human-astral	Orbitrap Astral	1	human	DIA
PXD034525	human-lumos	Orbitrap Fusion Lumos	16, 6 (GPF)	human	DIA
PXD028735	human-tripletof	TripleTOF 5600	3, 8 (GPF)	human	DIA
PXD028735	human-qe	Q Exactive HF-X	3, 6 (GPF)	human	DIA
PXD041421	human-timstof1	timsTOF Pro	4	human	DIA
PXD017703	human-timstof2	timsTOF Pro	3	human	DIA
PXD023325	100cell-eclipse	Orbitrap Eclipse	3	human	DIA
PXD023325	1cell-eclipse	Orbitrap Eclipse	3	human	DIA
PXD012988	mouse-qe	Q Exactive HF	15	mouse	DIA
MSV000084000	yeast-lumos	Orbitrap Fusion Lumos	3, 6 (GPF)	yeast	DIA

GPF: Gas-phase fractionation DIA.
