# Supplementary material for: Assessment of false discovery rate control in tandem mass spectrometry analysis using entrapment
Source: bioRxiv. 2024 Jun 4:2024.06.01.596967. Preprint. [Version 2] doi: 10.1101/2024.06.01.596967 (PMC11185562; doi:10.1101/2024.06.01.596967)
Supplement: Supplementary file 1 [file media-1.pdf]

Supplement to "Assessment of false discovery rate control in tandem mass spectrometry analysis using entrapment"

Bo Wen[1], Jack Freestone[2], Michael Riffle[1], Michael J. MacCoss[1], William S. Noble[1,3], and Uri Keich[2]

[1]Department of Genome Sciences, University of Washington
[2]School of Mathematics and Statistics, University of Sydney
[3]Paul G. Allen School of Computer Science and Engineering, University of Washington

S1 The combined entrapment method tends to overestimate the true FDP and the lower bound underestimates it

The underlying assumption of the combined entrapment estimation method is that because the entrapment database is r times larger than the sample database, a spectrum that is foreign to the combined database is r times more likely to be optimally matched to the entrapment than to the original target part of the combined database. In particular, in the extreme case that none of the original target peptides is present in the sample $N_{\mathcal{E}}$ is an unbiased estimator of $r \cdot V_T$, so in this case, the numerator of (1), $N_{\mathcal{E}}(1 + 1/r)$, is an unbiased estimator of $V_{\mathcal{E}} + V_T$, the number of false discoveries in the combined database.

Of course, in the typical case a subset $\mathcal{T}_p$ of the original target database, $\mathcal{T}$, is present in the sample. With $\mathcal{T}_m$ denoting the complementary set of those peptides that are not in the sample, we can split the entrapment dataset $\mathcal{E}_{\mathcal{T}}$ into two corresponding sets $\mathcal{E}_p$ and $\mathcal{E}_m$ so that each of those parts is roughly r times larger than the corresponding original target part.

The above discussion shows that $N_{\mathcal{E}_m}$, the number of entrapment discoveries in the subset $\mathcal{E}_m$, is an unbiased estimator of $r \cdot N_{\mathcal{T}_m} = r \cdot V_{\mathcal{T}_m}$, where $N_{\mathcal{T}_m}$ is the number of (false) original target discoveries in $\mathcal{T}_m$. At the same time $N_{\mathcal{E}_p} = V_{\mathcal{E}_p}$ is obviously an overestimate of $r \cdot V_{\mathcal{T}_p}$, because the latter is 0 by definition: all the peptides in $\mathcal{T}_p$ are in the sample so none can be falsely detected, even if the PSM that identifies it happens to be incorrect.

It follows that

$$EN_{\mathcal{E}} = EN_{\mathcal{E}_m} + EN_{\mathcal{E}_p} = EV_{\mathcal{E}_m} + EV_{\mathcal{E}_p} \geq r \cdot EV_{\mathcal{T}_m} = r \cdot EV_{\mathcal{T}}.$$

Therefore,

$$E\left[N_{\mathcal{E}} \cdot (1 + 1/r)\right] = EV_{\mathcal{E}} + EN_{\mathcal{E}} \cdot 1/r \geq EV_{\mathcal{E}} + EV_{\mathcal{T}},$$

showing the numerator in (1) indeed generally overestimates $V_{\mathcal{E}} + V_{\mathcal{T}}$, the number of false discoveries in the target+entrapment list of discoveries.

At the same time it is clear that

$$N_{\mathcal{E}} = V_{\mathcal{E}} \leq V_{\mathcal{E}} + V_{\mathcal{T}},$$

so (2) indeed a lower bound on the FDP in the target+entrapment discovery list.

S2 The sample entrapment method can underestimate or overestimate the FDP

The sample entrapment estimation method (3) aims to estimate the FDP only among the original target discoveries rather than among the combined list of target+entrapment discoveries. This approach is inherently problematic because the analyzing tool is asked to control the FDR in the target+entrapment list of discoveries while the evaluation is done only on the original target discoveries. As a result, while as explained next, the sample entrapment method typically underestimates the FDP, it can also potentially overestimate the FDP. Therefore, this method cannot be used to show that a tool controls the FDR, nor that it does not control the FDR. The related fallacy of trying to control the FDR in a subset by controlling it in the larger set has already been pointed out in the literature [2, 5].

Indeed, typically the FDP among the discoveries from the target $\mathcal{T}$ will be significantly lower than among the target+entrapment list of discoveries, and hence (3) will typically underestimate the FDP among the latter discoveries. Critically, it is the latter FDP that the search tool is actually trying to control, so typically the sample estimation will underestimate the true FDP.

To demonstrate that (3) can in some extreme cases overestimate the FDP, we find it simpler to assume that the analyzing tool aims to report peptides in DDA data. Consider a hypothetical case, where every original target peptide in $\mathcal{T}$ produces a very good spectrum that creates a perfect PSM. Suppose that, in addition to those perfect spectra, the dataset also contains a large number of other spectra that are foreign to $\mathcal{T}$. Because the native spectra will mostly be optimally matched to the original target peptides they were generated from, the entrapment peptides will overwhelmingly be optimally matched to the foreign spectra. Inevitably, some of those entrapment peptides will score fairly high and make it to the target+entrapment discovery list. Consequently, we will end up with $N_{\mathcal{E}} > 0$ and hence $\widehat{\text{FDP}}_{\mathcal{T}} > 0$ in (3), but the true FDP is 0 because there are no false sample matches, so (3) overestimates the true FDP in this case.

Other versions of (3) that aim to estimate the FDP among the discoveries from the original target $\mathcal{T}$ have been used as well. For example, Yu *et al.* [7] estimate this FDP by $\frac{N_{\mathcal{E}}}{N_{\mathcal{T}}} \frac{M_{\mathcal{T}} - n_{\mathcal{T}}}{M_{\mathcal{E}}}$, where $M_{\mathcal{T}}$ and $M_{\mathcal{E}}$ are the number of peptides in the original target and entrapment databases. Much of the above criticism applies to this estimator as well: it can overestimate as well as underestimate the FDP. For example, if the tool reports that it detected all the original target peptides, then this estimate is 0, but of course, this will typically underestimate the FDP. Similarly, if all the original target peptides are present in the sample then the actual FDP is 0, but this estimate will typically be positive.

S3 Potential pitfalls of using foreign entrapment sequences

Critical to all methods of FDP estimation is the ratio r of entrapment-to-original-target sequences. When using shuffled entrapment every original target sequence creates exactly r shuffled entrapment sequences, each with the same composition as the target's, and therefore there is no ambiguity about that ratio. In contrast, consider using a foreign entrapment in peptide-level analysis. The natural choice for r is the ratio of the number of entrapment-to-original-target peptides. However, it is conceivable that the entrapment peptides would have very different amino acid composition and length distributions compared with those of the original target peptides. Such differences can cast doubts over the adequacy of that simple ratio.

S3.1 Selecting the "right" evolutionary distance

An issue related to the last point is the critical selection of the foreign species: choose species that are at a very large evolutionary distance to your target species and you risk creating entrapment sequences that do not offer a realistic competition to the incorrect target discoveries they need to model. In this case you are likely to underestimate the true FDP when using the combined estimation. Conversely, if your entrapment includes a species that is a close relative of your target, then you run the risk of overestimating the FDP, particularly when considering sample peptides whose exact matches are missing from the original target database, say due to unexpected modifications or genetic variations.

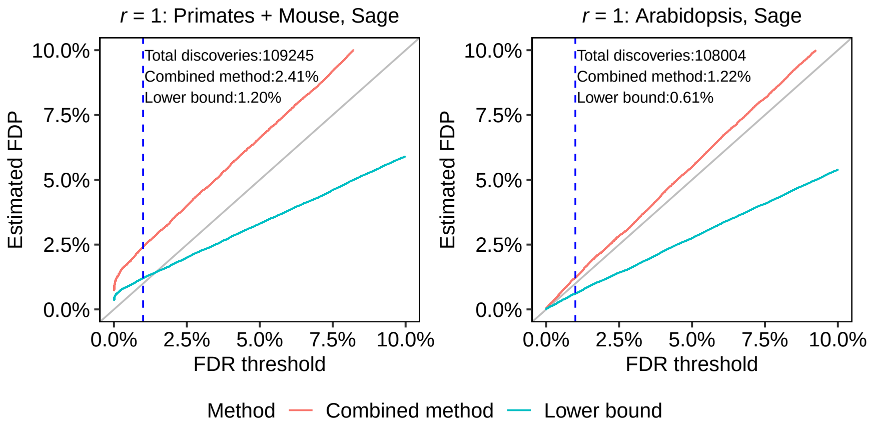

Figure S1: **Varying the evolutionary distance of the peptide-level foreign entrapment with Sage applied to the HEK293 dataset ($r = 1$).** The dashed vertical lines are at the 1% FDR threshold, as are the numbers reported in text in the panels.

We next demonstrate this point through a few sets of entrapment experiments we conducted, where in each set we use the same analysis tool and the same dataset, while varying the evolutionary distance of the foreign entrapment sequences even as we keep a fixed $r = 1$.

Figure S1 presents the different conclusions we can draw when evaluating Sage's peptide-level analysis of the human-sampled HEK293 DDA dataset using two different foreign entrapment resources (both described in Section 4.4). As expected, when using a mixture of (non-ape) primates and mouse proteins for our foreign entrapment (left panel), Sage's estimated FDPs are substantially higher than when using Arabidopsis for the entrapment (right panel). Because we cannot use the paired estimation when using a foreign entrapment, the combined estimation is the only way to establish FDR control in this case. However, the combined curve is noticeably above the diagonal in the left panel. In particular, it estimates the FDP for the 1% FDR threshold at a rather high 2.4%, thus calling into question Sage's FDR control had we only used that entrapment experiment. In contrast, using the Arabidopsis peptides the combined method is only marginally above the diagonal (right panel). Recalling the general conservative nature of the combined method this raises the possibility that using now the much more evolutionary distant Arabidopsis we might be slightly underestimating the FDP in this case.

We observe the same trends when using foreign entrapments to evaluate DIA-NN's analysis of the human-sampled PXD042704 (human-astral) DIA dataset. Specifically, Supplementary Figure S2's comparison of using the Primates+Mouse vs. Arabidopsis entrapment sequences to evaluate DIA-NN's precursor-level analysis looks qualitatively very similar to the corresponding peptide-level analysis of Sage in Figure S1, so the same comments apply here.

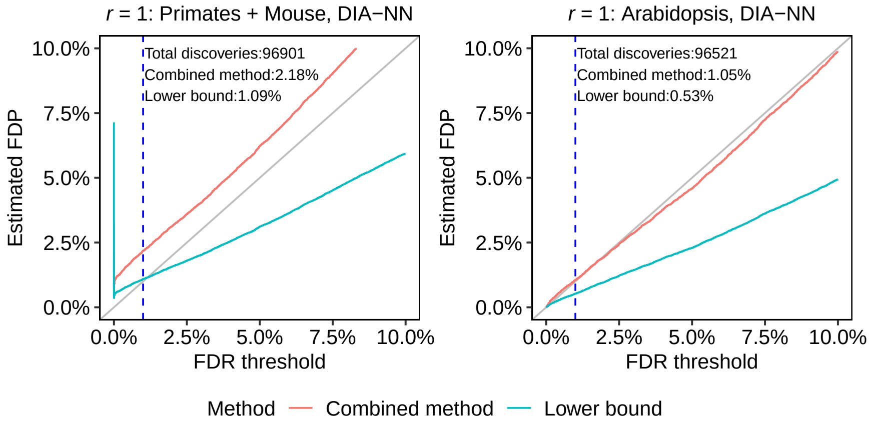

Figure S2: **Varying the evolutionary distance of the precursor-level foreign entrapment with DIA-NN applied to the DIA dataset PXD042704 (human-astral) with $r = 1$.** The dashed vertical lines are at the 1% FDR threshold, as are the numbers reported in text in the panels.

Similarly, the protein-level entrapment analyses depicted in Figure S3 again show that all estimated FDPs increase as the evolutionary distance to the human sample decreases.

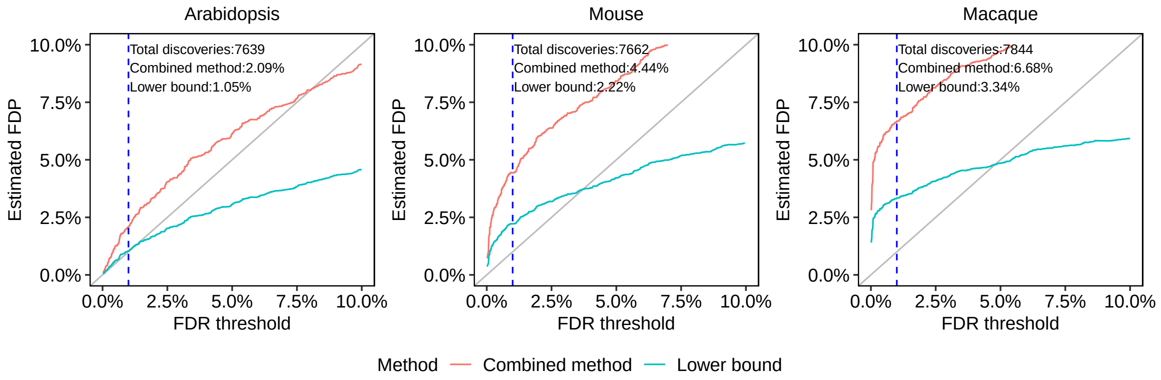

Figure S3: **Varying the evolutionary distance of the protein-level foreign entrapment with DIA-NN applied to the DIA dataset PXD042704 (human-astral) with $r = 1$.** The dashed vertical lines are at the 1% FDR threshold, as are the numbers reported in text in the panels.

S3.2 Foreign entrapments can be compromised by contamination

Finally, it is important to note that an entrapment experiment that relies on foreign sequences can be led astray by contamination. Indeed, compare the results of the two entrapment experiments summarized in Figure S4, where in both cases Sage peptide-level analysis was applied to the HEK293 dataset using two different foreign entrapments, both constructed so that $r = 1$: one made of yeast + Arabidopsis peptides (left panel) and the other made of Arabidopsis-only peptides (right panel). Clearly, adding the yeast peptides to the entrapment database significantly increases both the combined and the lower bound estimates of the FDP.

Looking more closely, we find that at the 1% FDR threshold Sage reported 108,867 peptides, of which 1,409 were entrapment peptides corresponding to a combined estimate of 2.59% ($r = 1$), and a lower bound of 1.29% on the FDP. Notably, 897 of the 1,409 discovered entrapment peptides (63.7%) were yeast peptides even though only 289,258 of the 1,389,436 entrapment peptides (20.8%) came from yeast. Statistically this is a highly significant enrichment of the yeast peptides among the discovered entrapment peptides (two sided Fisher Exact p-value $< 2.2e{-}16$). Unlike the examples in the previous section, yeast of course is not significantly closer to human than Arabidopsis, thus yeast contamination remains the most likely explanation.

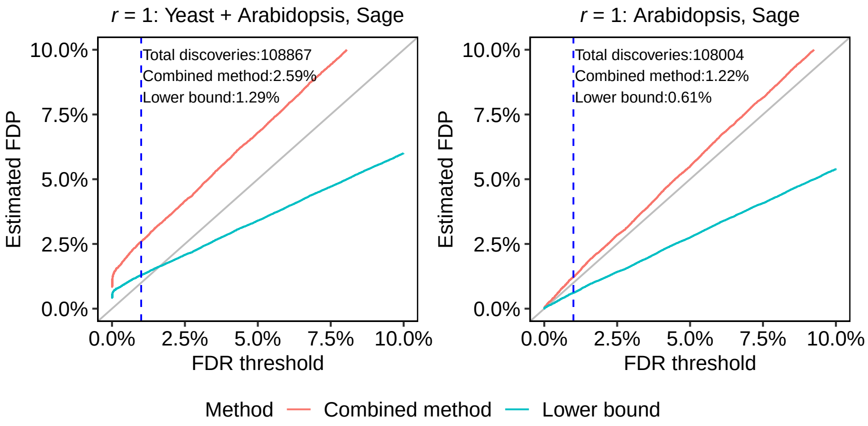

Figure S4: **Foreign entrapments can be biased by contamination.**

Consistently, searching the HEK293 MS/MS dataset against a protein database containing human and yeast proteins using Sage, we found that 1,510 out of the 111,376 peptides reported at 1% FDR match only to yeast proteins (a further 486 peptides match both the yeast and human proteins). Factoring in the ratio of human to yeast peptides this 1.4% of yeast peptides is much larger than expected. Moreover, if we specifically focus on the run with ID 02B, we find that 961 of the 9,390 peptides reported at 1% FDR match only to yeast proteins (a further 103 peptides match both the yeast and human proteins). This 10.2% yeast content is a fairly strong indication of some yeast contamination in that specific run.

S4 A survey of notable entrapment experiments

We conducted an extensive literature review looking at some prominent publications that used entrapment experiments. The results are summarized in Table 1, and here we discuss a few of those publications in more detail.

In [3] the authors estimated the FDP as $\frac{N_{\mathcal{E}} \cdot 1/r}{N_{\mathcal{T}} + N_{\mathcal{E}} \cdot 1/r}$, which does not seem to be well motivated. Applying this formula to the numbers they reported using—$N_{\mathcal{T}} = 8,867$, $N_{\mathcal{E}} = 297$ (*E. coli* peptides) and $r = 48,131/29,333$–we obtain the 0.02 estimated FDP that they reported. Regardless of the fact that this estimate is already higher than the 1% FDR threshold, if you use the combined estimation method, Equation (1), then you get an estimate higher than 0.05, or 5 times higher than the threshold. Moreover, even the lower bound, Equation (2), is larger than 0.03, or 3 times higher than the threshold, casting doubt over the claim that Specter controls the FDR.

In [4] the authors indicated that they used $r = 1.176$ and claim that the estimated FDPs (referred to as "external FDR") were in very good agreement with the corresponding thresholds ("internal FDR"). However, they used the sample method, which as we argued is invalid. Had the authors used the valid combined method, then taking r into account, the 1% FDP estimate they computed would have translated to 2.2%. Because this is not a lower bound it does not demonstrate MaxDIA fails to control the FDR, but at the same time this experiment does not provide evidence to support the fact that it controls the FDR.

In [7] the authors also used the invalid sample method to estimate the FDP among the quantified proteins reported by three different DIA tools. Using their reported values, $r = (6060 + 4401 + 16224)/20407 \approx 1.31$ the estimated FDPs (at 1% FDR) they reported for DIA-NN library-free, FP-MSF, and FP-MSF hybrid pipelines of 1.2%, 1.9%, and 1.8% respectively, correspond to 2.7%, 4.3% and 4.1% respectively if we use the valid combined method. Again, this analysis does not prove that these three tools do not control the FDR, but this experiment cannot be taken as evidence of proper FDR control.

In [1] the lower bound was correctly used to argue questionable FDR control in the case of MaxDIA, but it was also incorrectly used to argue proper FDR control of Spectronaut and DIA-NN. As we mentioned, the lower bound cannot be used as evidence of proper FDR control. For example, the authors report that using an *in silico* entrapment setup, DIA-NN's estimated FDP at 1% FDR was 1.77%, which they found acceptable. However, keeping in mind that this is a lower bound and that given that the entrapment part consisted of 16,202 *Arabidopsis* protein sequences and the original target was made of 17,082 mouse and 6,730 yeast protein sequences, we have $r \approx 0.68$. Therefore, using the same entrapment setup, the combined method estimates the FDP at 2.6%. The real FDP is somewhere in between, but it is highly questionable whether these results can be taken as evidence for proper FDR control.

The paper describing the recently released AlphaPept refers to some entrapment experiments, but the method is not fully described [6]. Looking at the accompanying code they released, we found that they were estimating the FDP as $N_{\mathcal{E}}/(N_{\mathcal{E}} + N_{\mathcal{E}} + N_{\mathcal{D}})$, where $N_{\mathcal{D}}$ is the number of decoys above the cutoff. This estimate is even smaller than the lower bound on the FDP (Equation (2)); hence, it cannot possibly be used as evidence of valid FDR control.

S5 Rationale behind the paired entrapment method and its extension

We argued in Supplementary Note S1 that, on average, the numerator in (1) overestimates the number of false discoveries. The paired entrapment estimation method can reduce this bias by taking advantage of the pairing information if it exists. Accordingly, in this section we require that our combined target+entrapment database contains pairs of original target and entrapment peptides (or proteins) $\{(t_i, e_i) : i = 1, \ldots, n\}$, so that $t_i \in \mathcal{T}_p$ if and only if $e_i \in \mathcal{E}_p$, and similarly for $\mathcal{T}_m$, and $\mathcal{E}_m$, where again, $\mathcal{T}_p$ and $\mathcal{T}_m$ are the original target peptides that are present, respectively, missing from the sample.

Noting that in this case the ratio of entrapment to original target size is $r = 1$, the above discussion in Section S1 shows that $N_{\mathcal{E}_m}$, the number of entrapment discoveries in the subset $\mathcal{E}_m$, is an unbiased estimator of $V_{\mathcal{T}_m}$. We can derive an alternative estimator of $V_{\mathcal{T}_m}$ by competing t_i with e_i as follows. Let s be the discovery cutoff score and let

$$N_{\mathcal{E}_m \geq s > \mathcal{T}_m} := |\{i : e_i \in \mathcal{E}_m, e_i \geq s > t_i\}| \qquad , \qquad N_{\mathcal{E}_m > \mathcal{T}_m \geq s} := |\{i : e_i \in \mathcal{E}_m, e_i > t_i \geq s\}| ,$$

where we can randomly break, or alternatively discard, pairs with $t_i = e_i$ (the results will slightly differ, but from a statistical perspective the two are equivalent). Note that we overload the notations t_i/e_i here to mean the peptides as well as their scores. We also define $N_{\mathcal{T}_m \geq s > \mathcal{E}_m}$ and $N_{\mathcal{T}_m > \mathcal{E}_m \geq s}$ by the obvious analogy with the roles of $\mathcal{E}_m$ and $\mathcal{T}_m$ swapped.

The combined estimation method relies on the fact that for $t_i \in \mathcal{T}_m$ and $r = 1$, $P(e_i \geq s) = P(t_i \geq s)$, and hence $N_{\mathcal{E}_m}$ is an unbiased estimator of $V_{\mathcal{T}_m}$. Examining the different contributions to $N_{\mathcal{E}_m}$ we find that

$$N_{\mathcal{E}_m} = N_{\mathcal{E}_m \geq s > \mathcal{T}_m} + N_{\mathcal{E}_m > \mathcal{T}_m \geq s} + N_{\mathcal{T}_m > \mathcal{E}_m \geq s}.$$

The equal chance assumption implies that for $t_i \in \mathcal{T}_m$ $P(e_i \geq s > t_i) = P(t_i \geq s > e_i)$, and that $P(e_i > t_i \geq s) = P(t_i > e_i \geq s)$. Therefore, $N_{\mathcal{E}_m \geq s > \mathcal{T}_m} + 2 \cdot N_{\mathcal{E}_m > \mathcal{T}_m \geq s}$ is an alternative unbiased estimator of

$$V_{\mathcal{T}_m} = N_{\mathcal{T}_m} = N_{\mathcal{T}_m \geq s > \mathcal{E}_m} + N_{\mathcal{T}_m > \mathcal{E}_m \geq s} + N_{\mathcal{E}_m > \mathcal{T}_m \geq s}.$$

In general, compared with $N_{\mathcal{E}_m}$, $N_{\mathcal{E}_m \geq s > \mathcal{T}_m} + 2 \cdot N_{\mathcal{E}_m > \mathcal{T}_m \geq s}$, is an inferior estimator of $V_{\mathcal{T}_m}$ because it considers less data than $N_{\mathcal{E}_m}$ does, and hence it has a higher variability. However, the advantage of the paired approach becomes clear once we consider the corresponding estimators of $V_{\mathcal{T}_p}$. Indeed, because $\mathcal{T}_p$ contains only peptides that are in the sample, we expect that for any reasonable scoring function and $t_i \in \mathcal{T}_p$, $P(e_i > t_i \geq s) < P(t_i > e_i \geq s)$, and therefore typically

$$N_{\mathcal{E}_p} = N_{\mathcal{E}_p \geq s > \mathcal{T}_p} + N_{\mathcal{E}_p > \mathcal{T}_p \geq s} + N_{\mathcal{T}_p > \mathcal{E}_p \geq s} > N_{\mathcal{E}_p \geq s > \mathcal{T}_p} + 2 \cdot N_{\mathcal{E}_p > \mathcal{T}_p \geq s}.$$

Because $V_{\mathcal{T}_p} = 0$ the fact that the estimator on the right hand side is generally smaller implies that it has a smaller bias.

The combined entrapment estimator (1) with $r = 1$ uses $N_{\mathcal{E}} = N_{\mathcal{E}_m} + N_{\mathcal{E}_p}$ to estimate $V_{\mathcal{T}}$, and hence $2N_{\mathcal{E}} = V_{\mathcal{E}} + N_{\mathcal{E}}$ to estimate $V_{\mathcal{E}} + V_{\mathcal{T}}$, the number of false target+entrapment discoveries. Similarly, our less conservative estimator is defined by adding $N_{\mathcal{E}_p \geq s > \mathcal{T}_p} + 2 \cdot N_{\mathcal{E}_p > \mathcal{T}_p \geq s}$ and $N_{\mathcal{E}_m \geq s > \mathcal{T}_m} + 2 \cdot N_{\mathcal{E}_m > \mathcal{T}_m \geq s}$ to estimate the same $V_{\mathcal{T}}$, and with $N_{\mathcal{E}} = V_{\mathcal{E}}$ as before we obtain (4).

The k-matched paired entrapment estimations method We can generalize the paired entrapment estimation method to use $k \geq 1$ entrapment peptides matched to each original target peptide, i.e., we assume the combined target+entrapment database contains $(k+1)$-tuples of an original target and k independently generated entrapment peptides that are associated with it: $\{(t_i, e_i^1, \ldots, e_i^k) : i = 1, \ldots, n\}$. In practice, we generate these k entrapment peptides by applying multiple shuffles to t_i and in particular $r = k$.

For $l = 1, \ldots, k$, let $N_{(l) \geq s}$ denote the number of original target peptides in $\mathcal{T}$ for which exactly l of its associated entrapment peptides score $\geq s$ and $t_i < \min\{s, e_i^1, \ldots, e_i^k\}$, i.e., the target scored worse than the threshold s as well as its k associated entrapments. Similarly, we let $N_{(k+1) \geq s}$ denote the number of original target peptides t_i that again scored worse than its k associated entrapments but $t_i \geq s$. We define the k-matched entrapment estimate as

$$\widehat{\text{FDP}}^{*k}_{\mathcal{T} \cup \mathcal{E}_{\mathcal{T}}} = \frac{N_{\mathcal{E}} + \sum_{l=1}^{k+1} l \cdot N_{(l) \geq s}}{N_{\mathcal{T}} + N_{\mathcal{E}}}. \tag{S1}$$

Clearly, (S1) reduces to (4) for $k = 1$, so the paired entrapment estimation (4) coincides with the $k = 1$ k-matched paired estimation. Moreover, it can be shown that for any reasonable scoring function for which for $t_i \in \mathcal{T}_p$, $P(e_i^j > t_i) < P(t_i > e_i^j)$ for all j, $\widehat{\text{FDP}}^{*k}_{\mathcal{T} \cup \mathcal{E}_\mathcal{T}}$ of (S1) would still overestimate the FDP, but less so than $\widehat{\text{FDP}}^{*}_{\mathcal{T} \cup \mathcal{E}_\mathcal{T}}$ of (4).

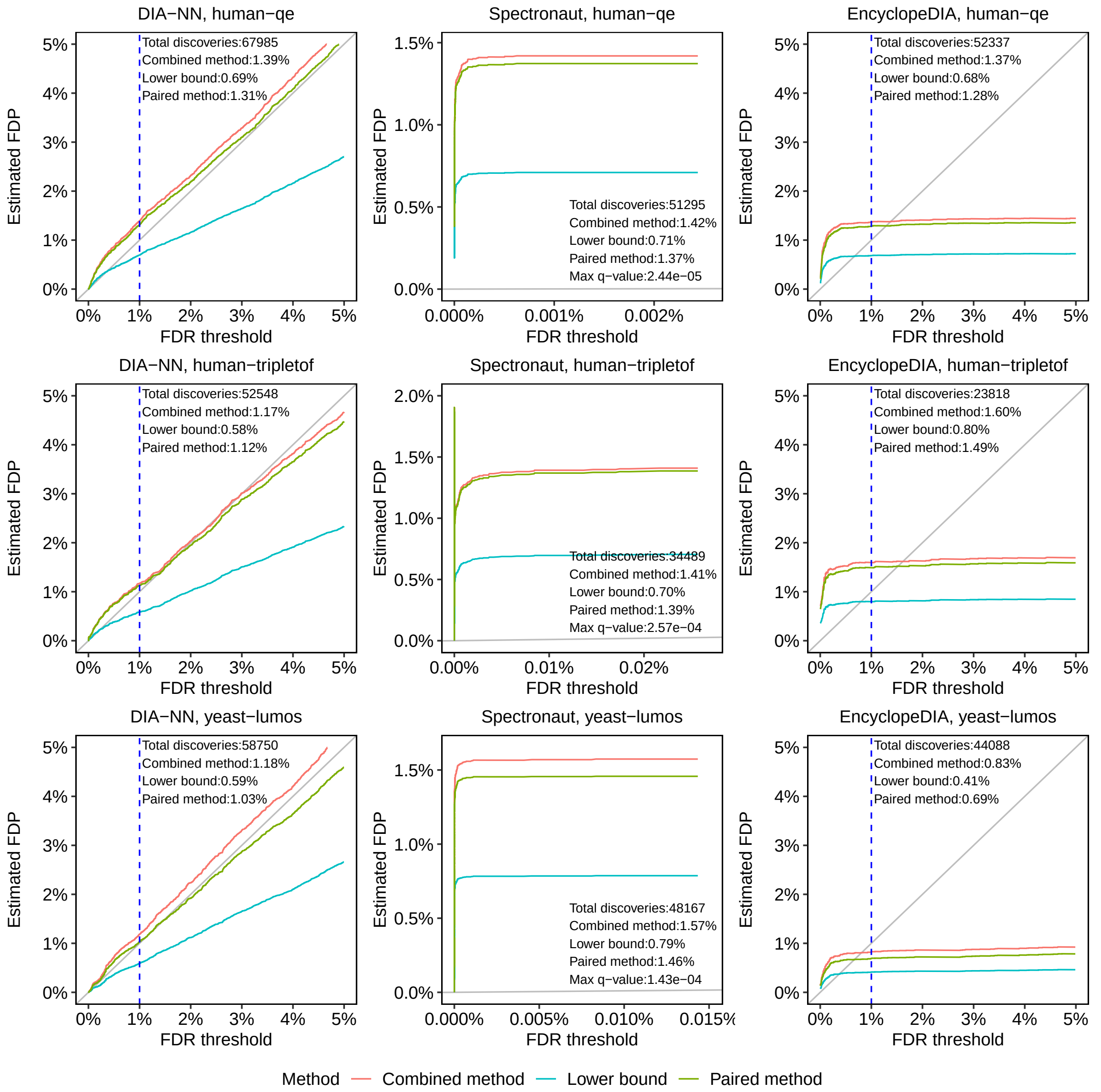

Figure S5: **Peptide or precursor-level FDR control evaluation of DIA search tools** In each panel, the empirical FDR was estimated for a given dataset (row) and search tool (column) using three different entrapment methods. Each panel plots the empirical FDR as a function of the FDR threshold. The dashed vertical lines are at the 1% FDR threshold, as are the numbers reported in text in the panels. The “Max q-value” value in each Spectronaut panel was the maximum reported q-value by the tool.

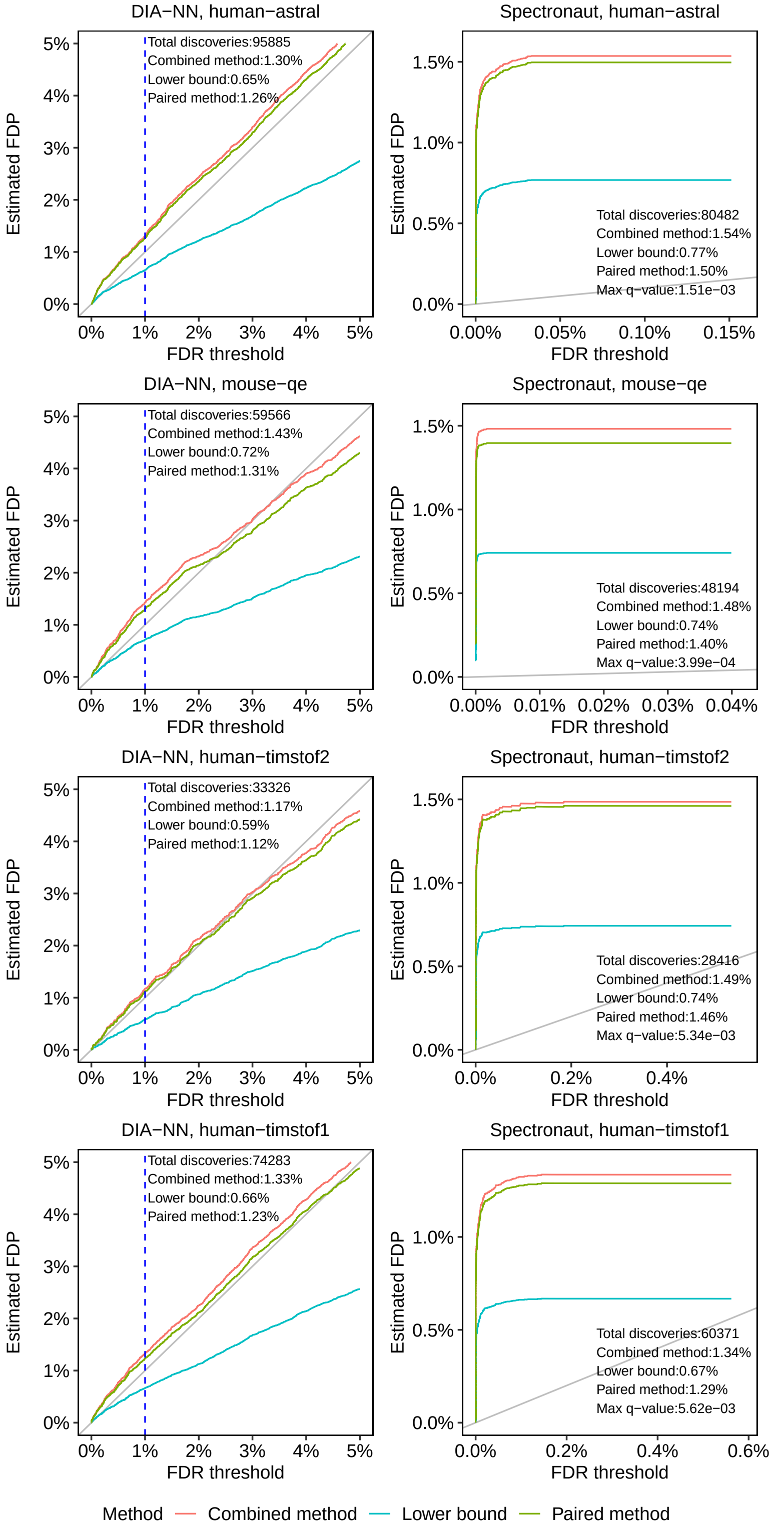

Figure S6: **Precursor-level FDR control evaluation of DIA search tools** In each panel, the empirical FDR was estimated for a given dataset (row) and search tool (column) using three different entrapment methods. Each panel plots the empirical FDR as a function of the FDR threshold. The dashed vertical lines are at the 1% FDR threshold, as are the numbers reported in text in the panels. The "Max q-value" value in each Spectronaut panel was the maximum reported q-value by the tool.

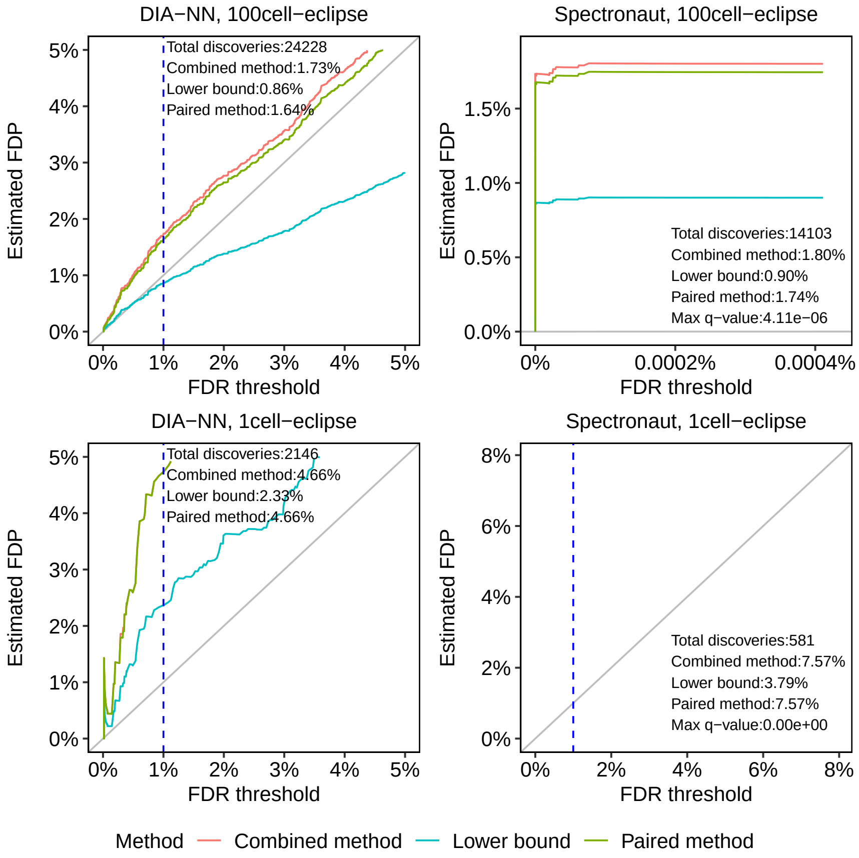

Figure S7: **Precursor-level FDR control evaluation of DIA search tools** In each panel, the empirical FDR was estimated for a given dataset (row) and search tool (column) using three different entrapment methods. Each panel plots the empirical FDR as a function of the FDR threshold. The dashed vertical lines are at the 1% FDR threshold, as are the numbers reported in text in the panels. The "Max q-value" value in each Spectronaut panel was the maximum reported q-value by the tool.

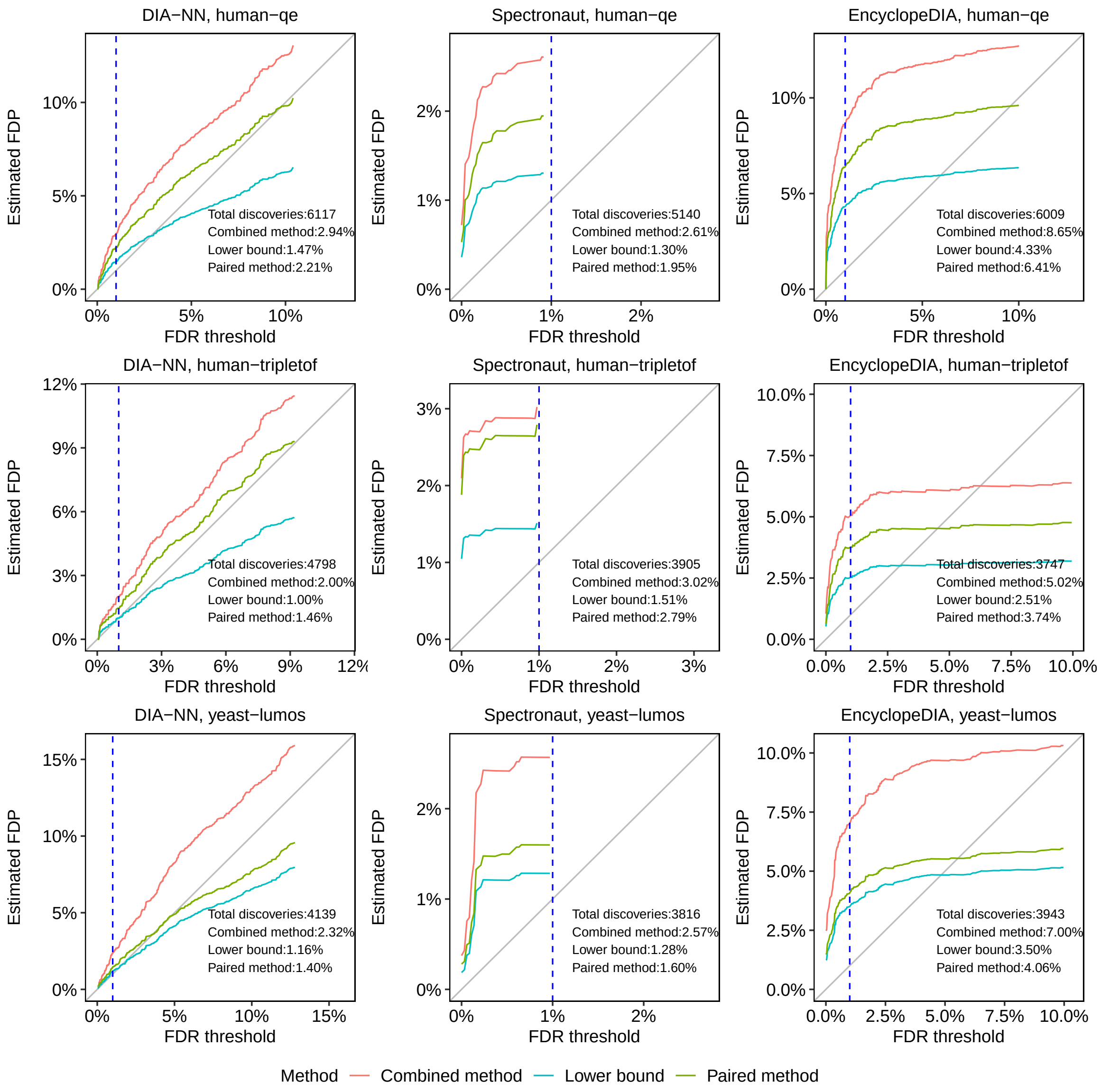

Figure S8: **Protein-level FDR control evaluation of DIA search tools** In each panel, the empirical FDR was estimated for a given dataset (row) and search tool (column) using three different entrapment methods. Each panel plots the empirical FDR as a function of the FDR threshold. The dashed vertical lines are at the 1% FDR threshold, as are the numbers reported in text in the panels.

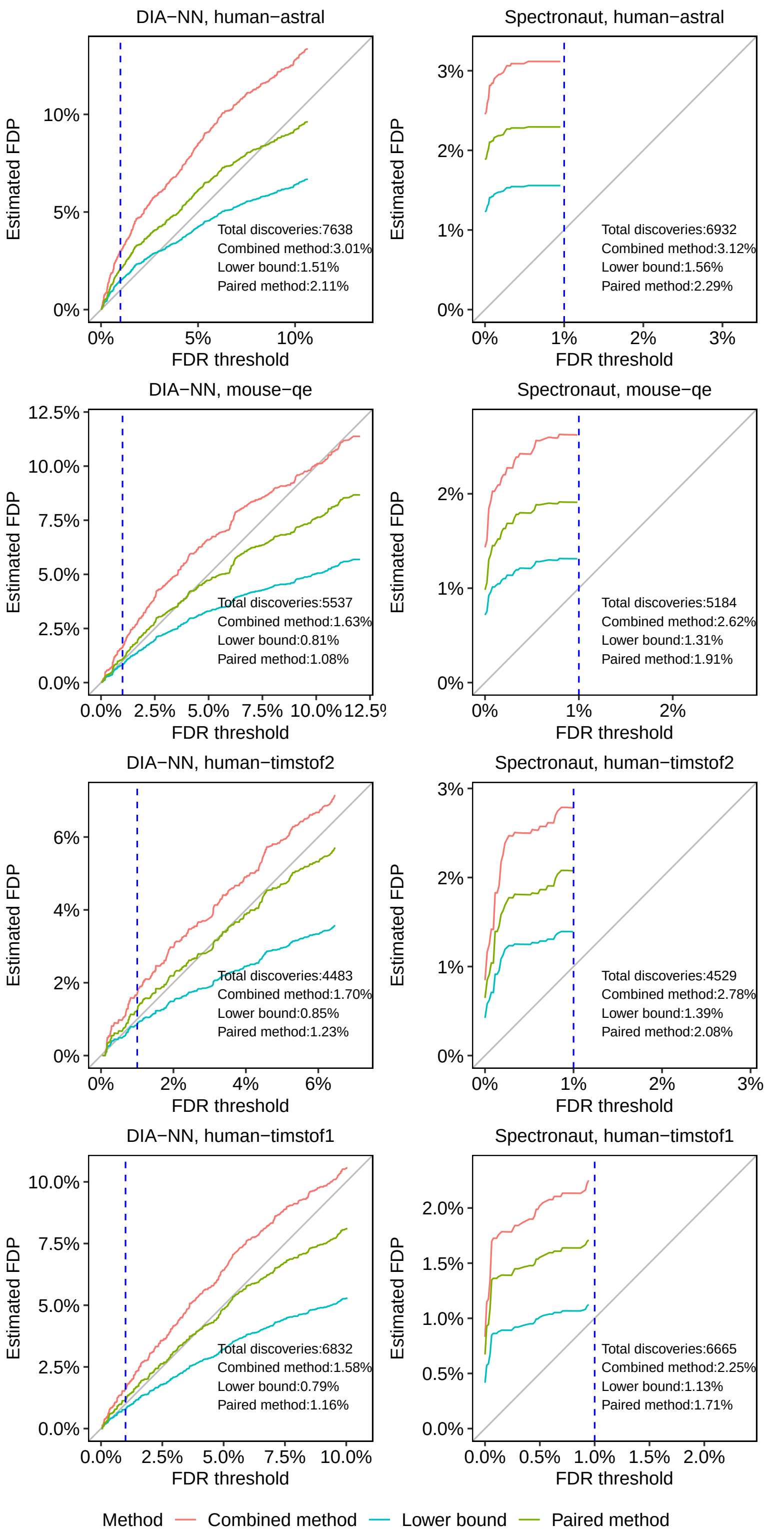

Figure S9: **Protein-level FDR control evaluation of DIA search tools** In each panel, the empirical FDR was estimated for a given dataset (row) and search tool (column) using three different entrapment methods. Each panel plots the empirical FDR as a function of the FDR threshold. The dashed vertical lines are at the 1% FDR threshold, as are the numbers reported in text in the panels.

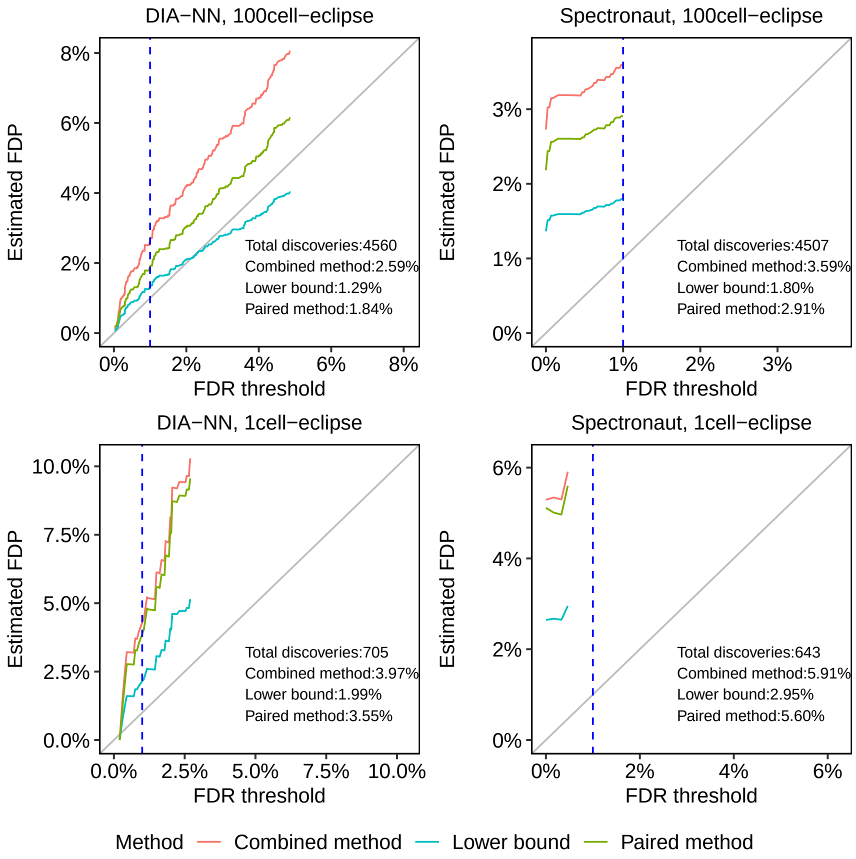

Figure S10: **Protein-level FDR control evaluation of DIA search tools** In each panel, the empirical FDR was estimated for a given dataset (row) and search tool (column) using three different entrapment methods. Each panel plots the empirical FDR as a function of the FDR threshold. The dashed vertical lines are at the 1% FDR threshold, as are the numbers reported in text in the panels.

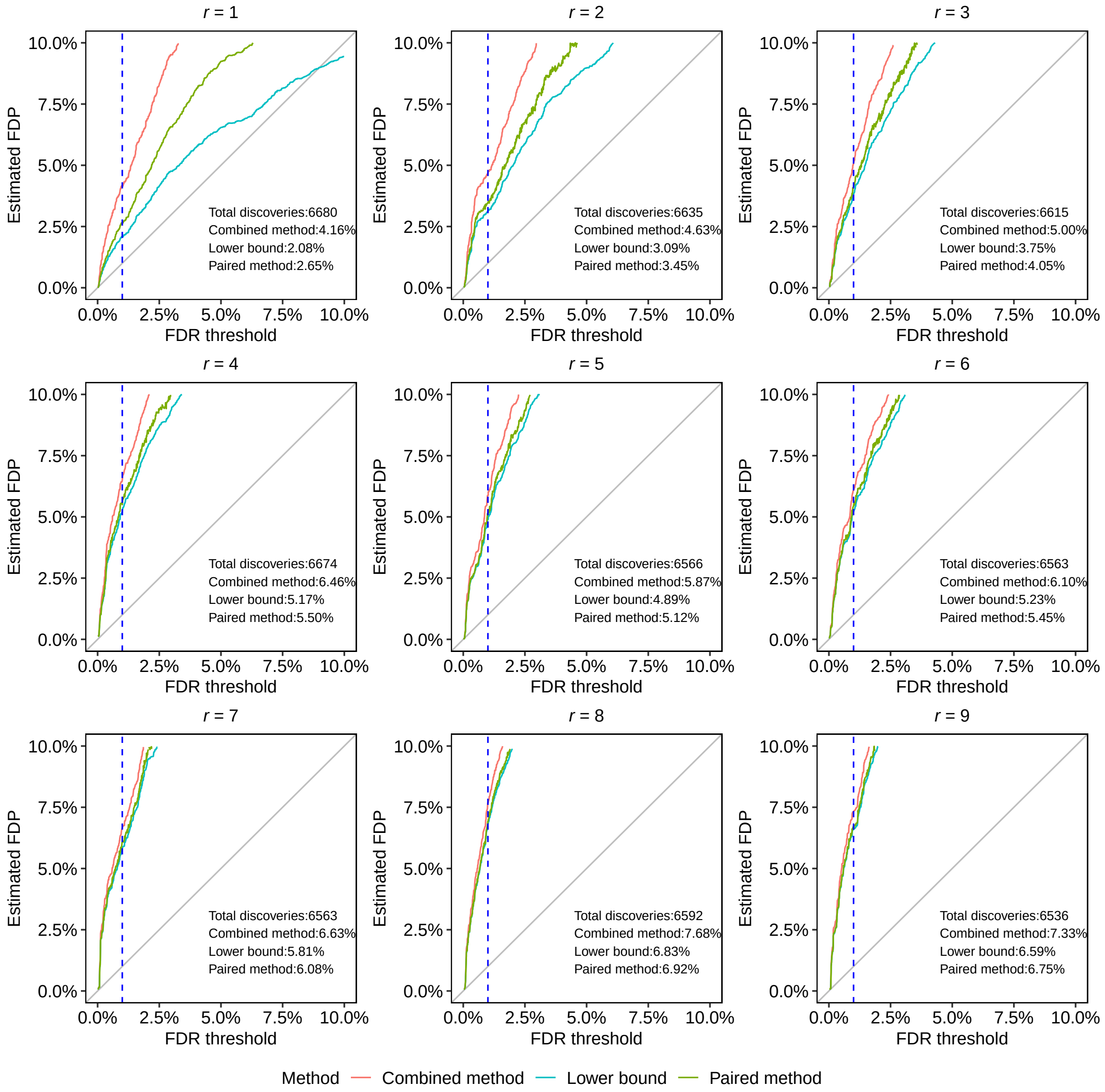

Figure S11: **DIA-NN struggles to control the FDR at the protein level as r increases on the DIA dataset PXD034525 (human-lumos).**

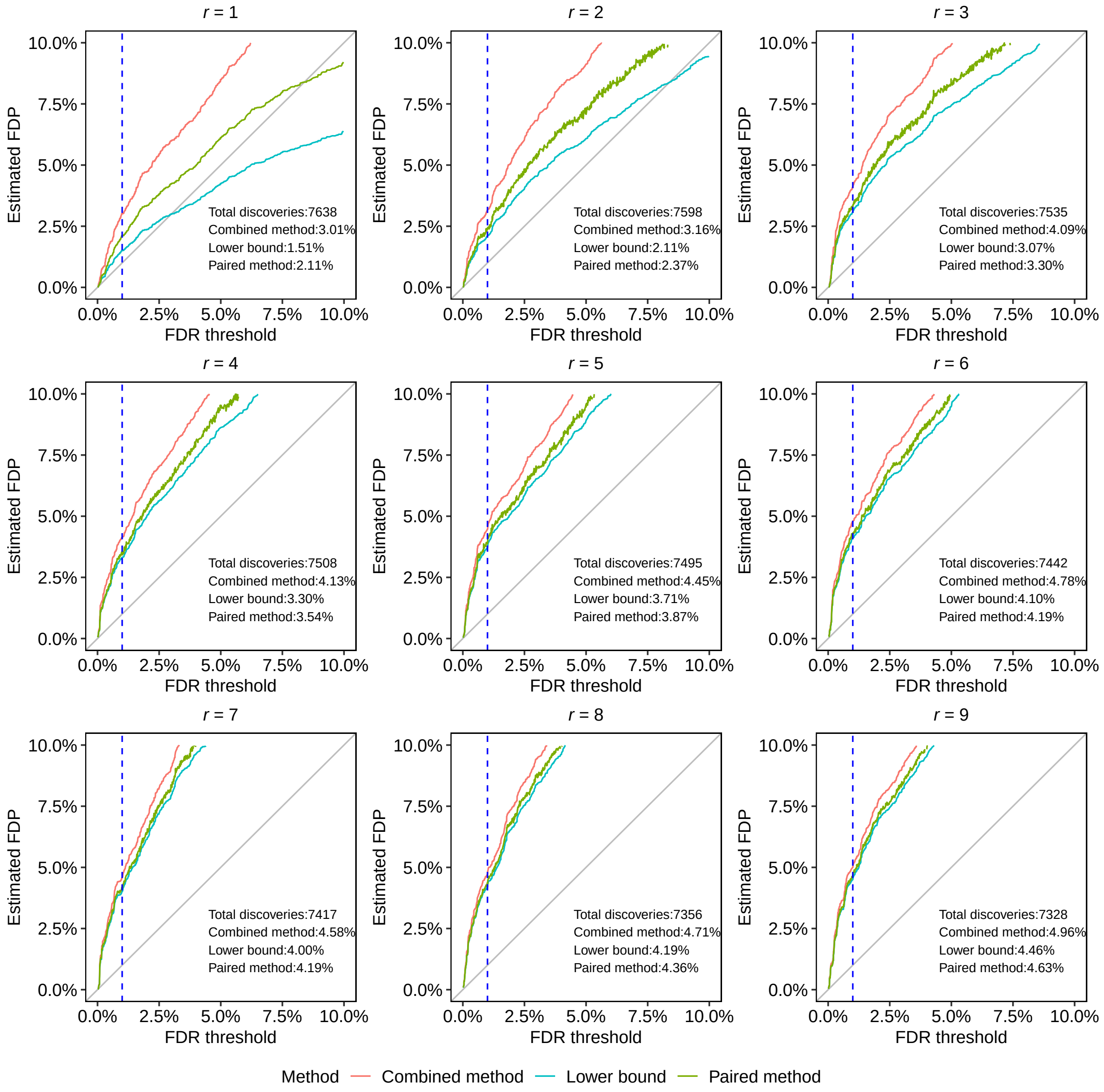

Figure S12: **DIA-NN struggles to control the FDR at the protein level as r increases on the DIA dataset PXD042704 (human-astral).**

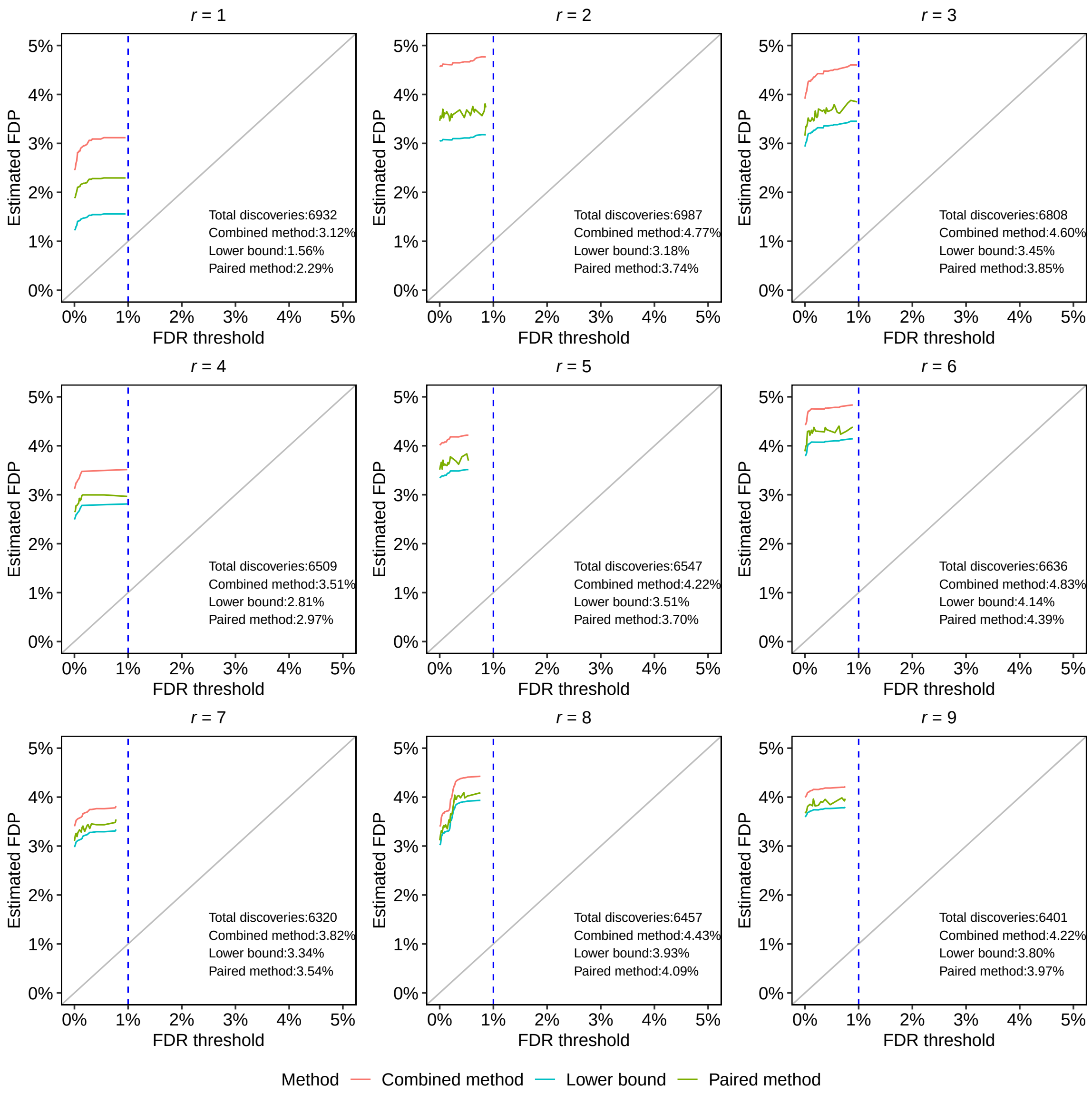

Figure S13: **Spectronaut struggles to control the FDR at the protein level as r increases on the DIA dataset PXD042704 (human-astral).** As r increases all methods report increasingly larger estimated FDP. Specifically, with $r = 6$ even the lower bound on the FDP among the 6,636 proteins discovered at 1% FDR is as high as 4.1%.

References

[1] R. Lou, Y. Cao, S. Li, X. Lang, Y. Li, Y. Zhang, and W. Shui. Benchmarking commonly used software suites and analysis workflows for dia proteomics and phosphoproteomics. *Nature Communications*, 14(1):94, 2023.

[2] W. S. Noble and U. Keich. Response to "Mass spectrometrists should search for all peptides, but assess only the ones they care about". *Nature Methods*, 14(7):644, 2017.

[3] R. Peckner, S. A. Meyers, J. D. Egertson, R. S. Johnson, J. G. Abelin, S. A. Carr, M. J. MacCoss, and J. D. Jaffe. Specter: linear deconvolution as a new paradigm for targeted analysis of data-independent acquisition mass spectrometry proteomics. *Nature Methods*, 15(5):371–378, 2018.

[4] P. Sinitcyn, H. Hamzeiy, F. Salinas Soto, D. Itzhak, F. McCarthy, C. Wichmann, M. Steger, U. Ohmayer, U. Distler, S. Kaspar-Schoenefeld, et al. MaxDIA enables library-based and library-free data-independent acquisition proteomics. *Nature Biotechnology*, 39(12):1563–1573, 2021.

[5] A. Sticker, L. Martens, and L. Clement. Mass spectrometrists should search for all peptides, but assess only the ones they care about. *Nature Methods*, 14(7):643–644, 2017.

[6] M. T. Strauss, I. Bludau, W.-F. Zeng, E. Voytik, C. Ammar, J. P. Schessner, R. Ilango, M. Gill, F. Meier, S. Willems, et al. AlphaPept: a modern and open framework for MS-based proteomics. *Nature Communications*, 15(1):2168, 2024.

[7] F. Yu, G. C. Teo, A. T. Kong, K. Fröhlich, G. X. Li, V. Demichev, and A. I. Nesvizhskii. Analysis of DIA proteomics data using MSFragger-DIA and FragPipe computational platform. *Nature Communications*, 14(1):4154, 2023.
